# Supplementary material for: HPF1/C4orf27 Is a PARP-1-Interacting Protein that Regulates PARP-1 ADP-Ribosylation Activity
Source: Mol Cell. 2016 May 5;62(3):432–42. doi: 10.1016/j.molcel.2016.03.008 (PMC4858568; doi:10.1016/j.molcel.2016.03.008)
Supplement: Document S1. Supplemental Experimental Procedures and Figures S1–S4 [file mmc1.pdf]

Molecular Cell, Volume *62*

Supplemental Information

HPF1/C4orf27 Is a PARP-1-Interacting Protein that Regulates PARP-1 ADP-Ribosylation Activity

Ian Gibbs-Seymour, Pietro Fontana, Johannes Gregor Matthias Rack, and Ivan Ahel

A

Organism	GenBank Accession no.	Function	Closest human homologue
Vertebrates	NP_775816	DNA repair	(APLF)
	NP_055696	DNA repair	(SNM1A)
	NP_001154816	DNA repair	(CHFR)
Drosophila melanogaster	NP_523465	DNA repair	TDP1
	NP_650455	DNA repair	APLF
	NP_649589	Unknown	C4orf27
Crassostrea gigas	XP_011423724	DNA repair	TDP1
	XP_011439503	DNA repair	APLF
	XP_011414049	Unknown	C4orf27
	XP_011433142	DNA repair	DNA ligase
	XP_011429878	(ADP-ribosyl)ation	PARP12
Dictyostelium discoideum	XP_628916	(ADP-ribosyl)ation	MACROD1
	XP_638785	(ADP-ribosyl)ation	TNKS2
	XP_641374	DNA repair	CHK2
	EAL61231	DNA repair	RAD17
	XP_637925	DNA repair	KU70
	XP_629877	DNA repair	UNG
	XP_641760	DNA repair	CHFR
Perkinsus marinum	XP_002765077	RNA recognition motifs (RRM) containing	CUGBP Elav-like family member 3
Emiliania huxleyi	XP_005762741	DNA repair	PNKP
	XP_005790986	Protease	Calpain-15
	XP_005780996	DNA repair	DNA ligase
Thecamonas trahens	XP_013754104	DNA repair	DNA ligase
	XP_013752674	Serine/threonine kinase	MAP3K7
	XP_013761104	DNA repair	Polβ
Capsaspora owczarzaki	XP_004365034	E2 ubiquitin-conjugating enzyme	UBE2Q1
	XP_004346784	Serine/threonine kinase	MAP3K7
	XP_004364110	DNA repair	DNA ligase
	XP_004364017	DNA repair	SNM1A

B

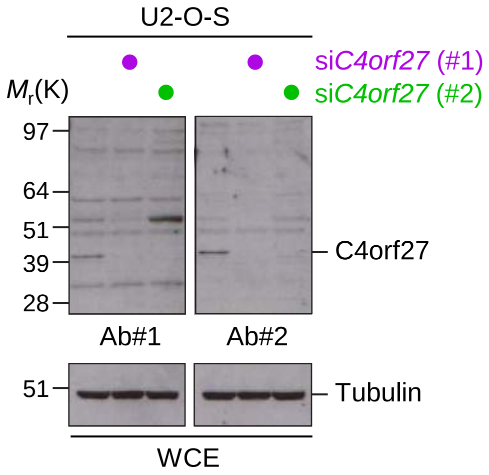

C

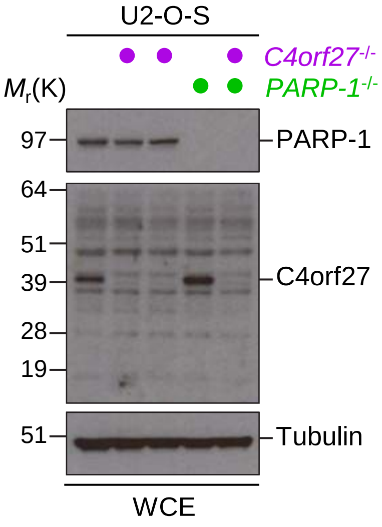

D

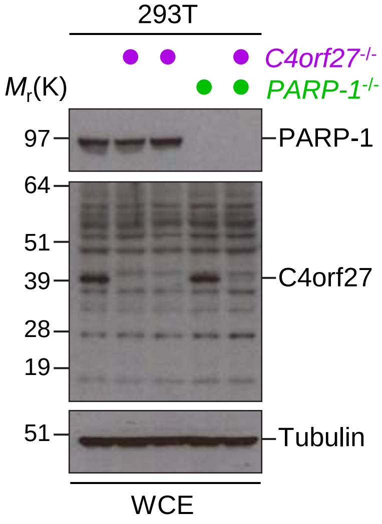

Figure S1 (related to Figure 1).

Identification of novel PBZ domain-containing proteins in eukaryotes

- **A.** Table showing PBZ-domain containing proteins in eukaryotes and their closest human orthologs.
- **B.** U2-O-S cells were transfected with siRNAs against C4orf27 for 72 h and whole cell extracts were analysed by immunoblotting with the indicated antibodies.
- **C.** U2-O-S cells with the indicated genetic backgrounds were generated using CRISPR/Cas9 and whole cell extracts were analysed by immunoblotting with the indicated antibodies.
- **D.** As for (C), but for 293T cells.

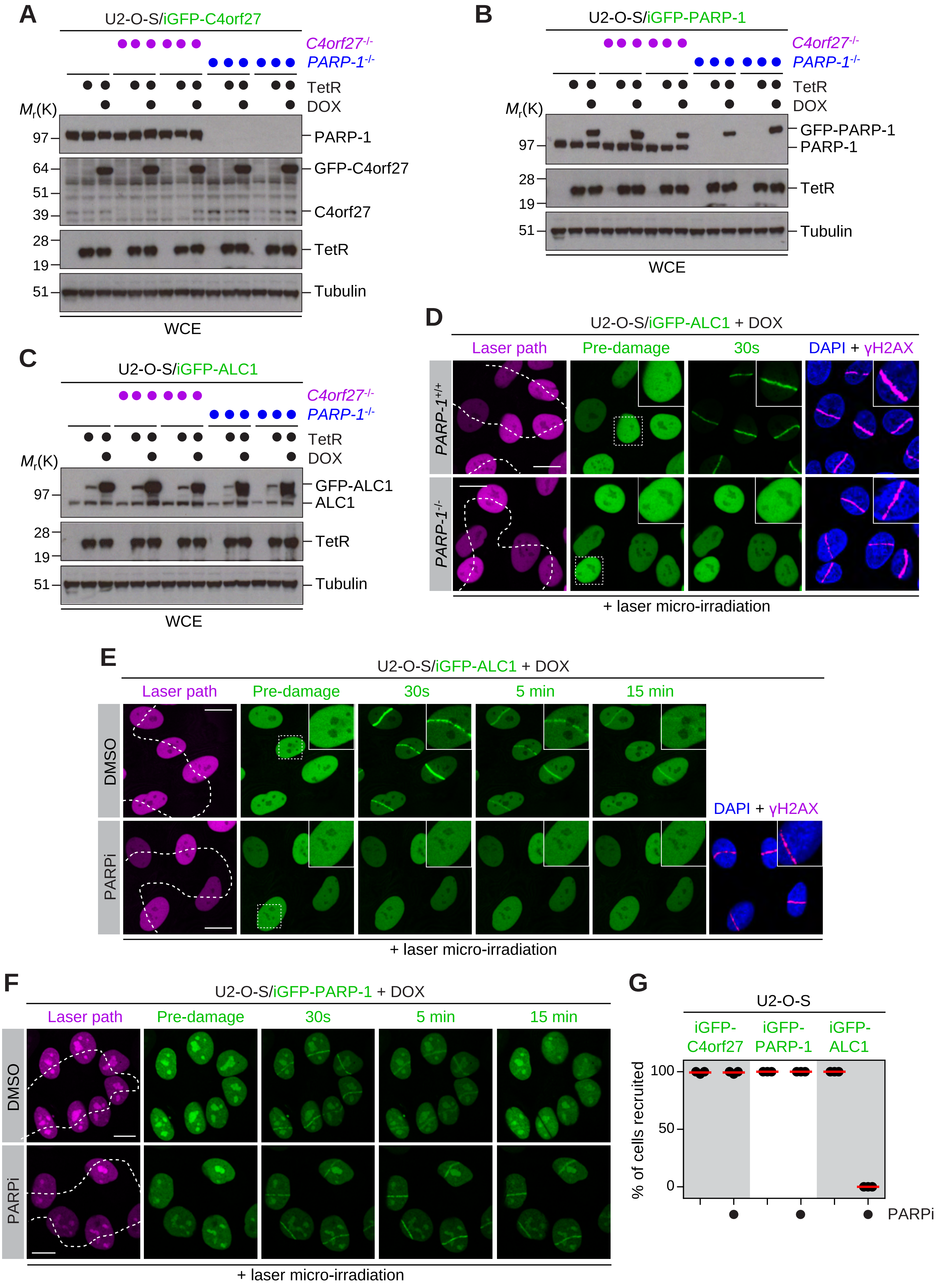
A
U2-O-S/iGFP-C4orf27
C4orf27-/-
PARP-1-/-
TetR
DOX
Mr(K)
97
64
51
39
28
19
51
PARP-1
GFP-C4orf27
C4orf27
TetR
Tubulin
WCE
B
U2-O-S/iGFP-PARP-1
C4orf27-/-
PARP-1-/-
TetR
DOX
Mr(K)
97
28
19
51
GFP-PARP-1
PARP-1
TetR
Tubulin
WCE
C
U2-O-S/iGFP-ALC1
C4orf27-/-
PARP-1-/-
TetR
DOX
Mr(K)
97
28
19
51
GFP-ALC1
ALC1
TetR
Tubulin
WCE
D
U2-O-S/iGFP-ALC1 + DOX
Laser path
Pre-damage
30s
DAPI + γH2AX
PARP-1+/+
PARP-1-/-
+ laser micro-irradiation
E
U2-O-S/iGFP-ALC1 + DOX
Laser path
Pre-damage
30s
5 min
15 min
DMSO
PARPi
DAPI + γH2AX
+ laser micro-irradiation
F
U2-O-S/iGFP-PARP-1 + DOX
Laser path
Pre-damage
30s
5 min
15 min
DMSO
PARPi
+ laser micro-irradiation
G
U2-O-S
iGFP-C4orf27
iGFP-PARP-1
iGFP-ALC1
% of cells recruited
100
50
0
PARPi

Figure S2 (related to Figure 2).

Generation of tools and laser micro-irradiation controls

A. Indicated iGFP-C4orf27 cell lines (two independent clones for the *C4orf27*$^{-/-}$ and *PARP-1*$^{-/-}$ backgrounds) were treated with DOX for 24 h and whole cell extracts were analysed by immunoblotting with the antibodies shown.

B. Indicated iGFP-PARP-1 cell lines (two independent clones for the *C4orf27*$^{-/-}$ and *PARP-1*$^{-/-}$ backgrounds) were treated with DOX for 24 h and whole cell extracts were analysed by immunoblotting with the antibodies shown.

C. Indicated iGFP-ALC1 cell lines (two independent clones for the *C4orf27*$^{-/-}$ and *PARP-1*$^{-/-}$ backgrounds) were treated with DOX for 24 h and whole cell extracts were analysed by immunoblotting with the antibodies shown.

D. U2-O-S/*PARP-1*$^{+/+}$ iGFP-ALC1 or U2-O-S/*PARP-1*$^{-/-}$ iGFP-ALC1 cells were induced with doxycycline (+DOX) for 24 h, subjected to laser micro-irradiation and imaged by live-cell microscopy at the indicated time. Cells were subsequently fixed and immunostained with γH2AX antibody. Scale bar, 10 µm.

E. U2-O-S/iGFP-ALC1 cells were induced with DOX for 24 h, pre-treated with DMSO or PARPi for 1 h and then subjected to laser micro-irradiation and imaged by live-cell microscopy at the indicated time. Cells were subsequently fixed and immunostained with γH2AX antibody. Scale bar, 10 µm.

F. U2-O-S/iGFP-PARP-1 cells were induced with DOX for 24 h, pre-treated with DMSO or PARPi for 1 h and then subjected to laser micro-irradiation and imaged by live-cell microscopy at the indicated time. Scale bar, 10 µm.

G. Quantification of laser micro-irradiation experiments from Fig 2E and Fig S2E,F. Data represents mean±SEM from three biologically independent experiments. At least 50 cells were quantified in each experiment.

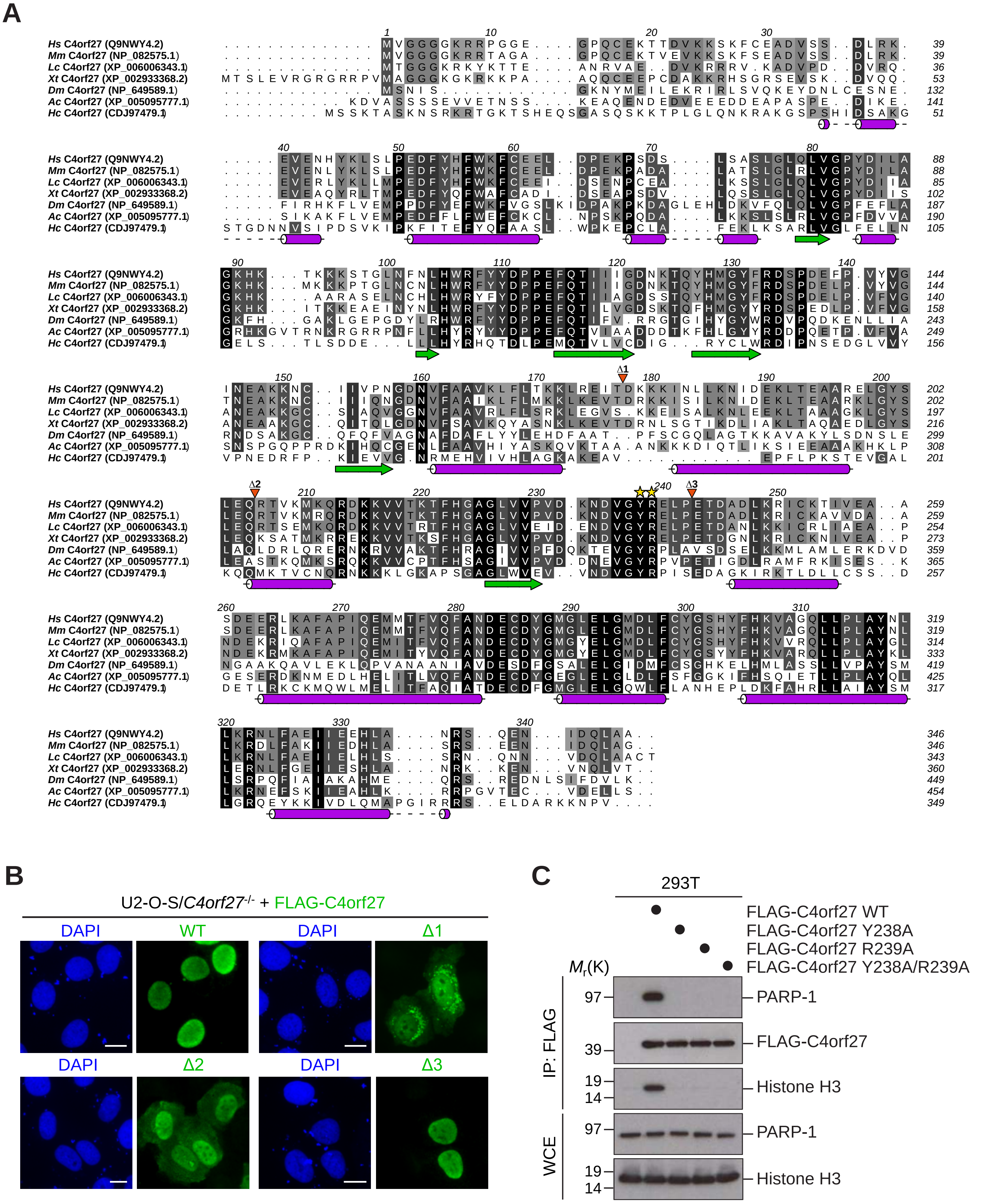
A
Hs C4orf27 (Q9NWY4.2)
Mm C4orf27 (NP_082575.1)
Lc C4orf27 (XP_006006343.1)
Xt C4orf27 (XP_002933368.2)
Dm C4orf27 (NP_649589.1)
Ac C4orf27 (XP_005095777.1)
Hc C4orf27 (CDJ97479.1)
Δ1
Δ2
Δ3
B
U2-O-S/C4orf27-/- + FLAG-C4orf27
DAPI
WT
DAPI
Δ1
DAPI
Δ2
DAPI
Δ3
C
293T
FLAG-C4orf27 WT
FLAG-C4orf27 Y238A
FLAG-C4orf27 R239A
FLAG-C4orf27 Y238A/R239A
Mr(K)
IP: FLAG
97
39
19
14
PARP-1
FLAG-C4orf27
Histone H3
WCE
97
19
14
PARP-1
Histone H3

Figure S3 (related to Figure 3).

Identification of residues important for the C4orf27-PARP-1 interaction

A. Sequence alignment and secondary structure prediction of human C4orf27 and selected orthologs. Alignment representation was created using the ALINE software (Bond and Schuttelkopf, 2009). The locations of the Δ1, Δ2 and Δ3 deletion mutants are denoted by orange triangles and residues important for the PARP-1 interaction (Tyr238 and Arg239) are indictaed by yellow asterisks. *Hs, Homo sapiens*; *Mm, Mus musculus*; *Lc, Latimeria chalumnae*; *Xt*, *Xenopus tropicalis*; *Dm*, *Drosophila melanogaster*; *Ac, Aplysia californica*; *Hc*, *Haemonchus contortus*.

B. U2-O-S/*C4orf27*$^{-/-}$ cells were transfected with FLAG-C4orf27 WT or Δ1, Δ2 or Δ3 deletion mutants, fixed and immunostained with anti-FLAG antibody. Scale bar, 10 µm.

C. 293T cells were transfected with either FLAG-empty vector, FLAG-C4orf27 WT or point mutants Y238A, R239A or Y238A/R239A, subjected to FLAG immunoprecipitation and analysed by immunoblotting with the indicated antibodies.

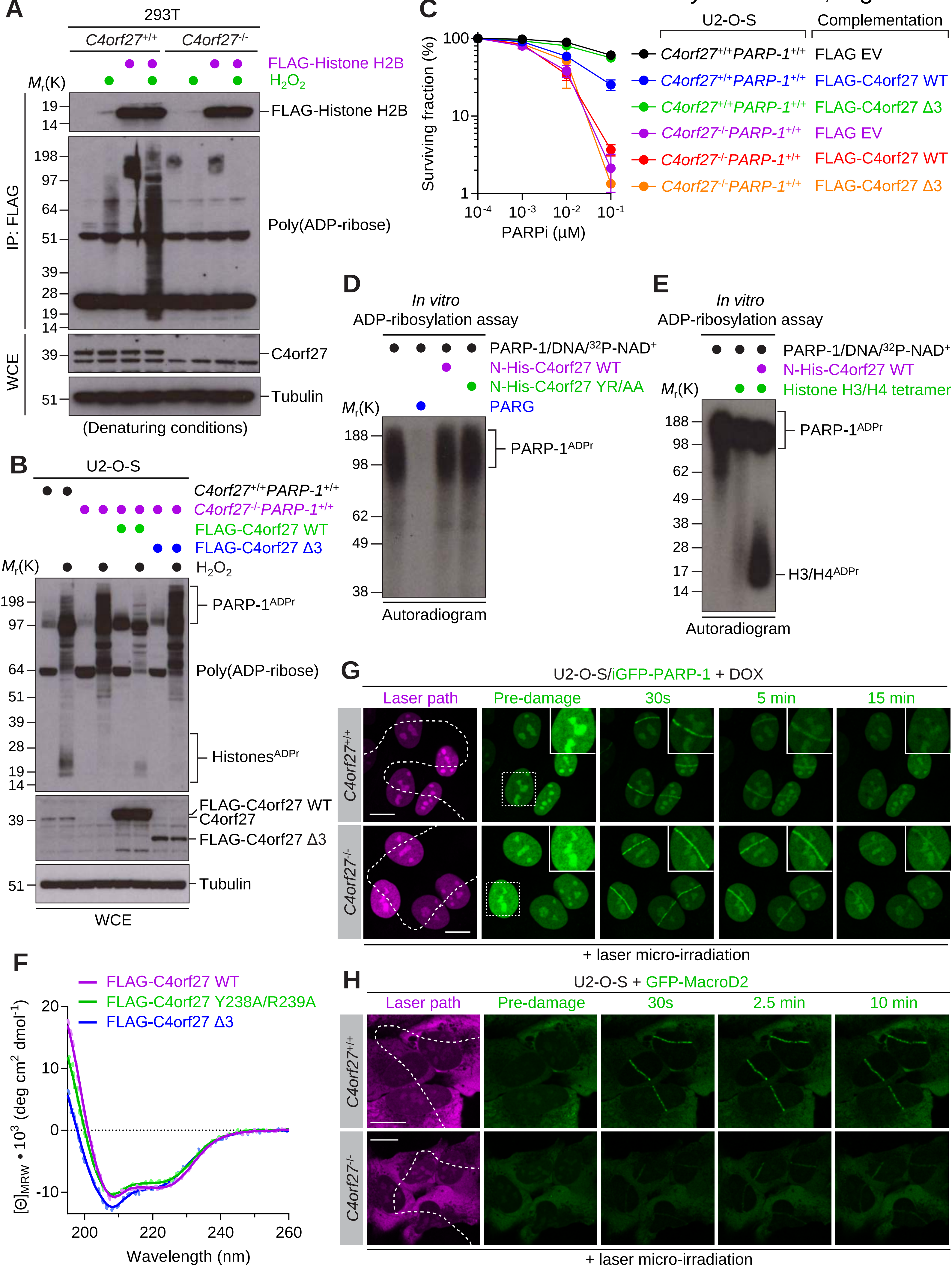
A
293T
C4orf27+/+
C4orf27-/-
FLAG-Histone H2B
H2O2
Mr(K)
IP: FLAG
FLAG-Histone H2B
Poly(ADP-ribose)
WCE
C4orf27
Tubulin
(Denaturing conditions)
B
U2-O-S
C4orf27+/+PARP-1+/+
C4orf27-/-PARP-1+/+
FLAG-C4orf27 WT
FLAG-C4orf27 Δ3
H2O2
PARP-1ADPr
Poly(ADP-ribose)
HistonesADPr
FLAG-C4orf27 WT
C4orf27
FLAG-C4orf27 Δ3
Tubulin
WCE
C
U2-O-S
Complementation
Surviving fraction (%)
PARPi (μM)
C4orf27+/+PARP-1+/+ FLAG EV
C4orf27+/+PARP-1+/+ FLAG-C4orf27 WT
C4orf27+/+PARP-1+/+ FLAG-C4orf27 Δ3
C4orf27-/-PARP-1+/+ FLAG EV
C4orf27-/-PARP-1+/+ FLAG-C4orf27 WT
C4orf27-/-PARP-1+/+ FLAG-C4orf27 Δ3
D
In vitro
ADP-ribosylation assay
PARP-1/DNA/32P-NAD+
N-His-C4orf27 WT
N-His-C4orf27 YR/AA
PARG
PARP-1ADPr
Autoradiogram
E
In vitro
ADP-ribosylation assay
PARP-1/DNA/32P-NAD+
N-His-C4orf27 WT
Histone H3/H4 tetramer
PARP-1ADPr
H3/H4ADPr
Autoradiogram
F
FLAG-C4orf27 WT
FLAG-C4orf27 Y238A/R239A
FLAG-C4orf27 Δ3
[Θ]MRW • 10³ (deg cm² dmol⁻¹)
Wavelength (nm)
G
U2-O-S/iGFP-PARP-1 + DOX
Laser path
Pre-damage
30s
5 min
15 min
C4orf27+/+
C4orf27-/-
+ laser micro-irradiation
H
U2-O-S + GFP-MacroD2
Laser path
Pre-damage
30s
2.5 min
10 min
C4orf27+/+
C4orf27-/-
+ laser micro-irradiation

Figure S4 (related to Figure 4).

C4orf27 promotes *in trans* ADP-ribosylation of histones and prevents PARP-1 hyper auto-modification

- **A.** 293T/*C4orf27*$^{+/+}$ or 293T/*C4orf27*$^{-/-}$ cells were transfected with FLAG-empty vector, or FLAG-histone H2B, treated with either vehicle or H_2O_2 (1 μM) for 10 min, lysed under denaturing conditions and then subjected to FLAG immunoprecipitation and analysed by immunoblotting with the indicated antibodies.
- **B.** U2-O-S cells with the indicated genetic backgrounds were treated with H_2O_2 (1 μM) for 10 min and whole cell extracts were analysed by immunoblotting with the indicated antibodies. ADP-ribosylated PARP-1 (PARP-1^{ADPr}) and histones (Histones^{ADPr}) are indicated.
- **C.** U2-O-S/*C4orf27*$^{+/+}$ or U2-O-S/*C4orf27*$^{-/-}$ cells complemented with empty vector, FLAG-C4orf27 WT or FLAG-C4orf27 Δ3 were treated with various doses of PARPi and analysed for clonogenic survival. Data represents mean±SEM from three biologically independent experiments using technical triplicates per datapoint.
- **D.** Recombinant PARP-1 was incubated with DNA and ^{32}P-NAD^{+} to produce ADP-ribosylated PARP-1, before the addition of PARP inhibitor to stop the reaction. Recombinant PARG or C4orf27 was then added to the reaction and products were analysed by SDS-PAGE and autoradiography.
- **E.** Recombinant PARP-1 and N-His-C4orf27 were incubated with recombinant histone H3/H4 tetramer and reactions were initiated by addition of DNA and ^{32}P-NAD^{+}. Reaction products were subsequently analysed by SDS-PAGE and autoradiography.
- **F.** CD analysis of recombinant C4orf27 wild type, Y238A/R239A and Δ3. CD spectra (195-260nm) were obtained at 20°C and buffer corrected. Dashed lines represent

accumulated spectra measurements (*n*=15) and solid lines smoothed spectra using the Savitzky-Golay algorithm.

G. U2-O-S/*C4orf27*$^{+/+}$ iGFP-PARP-1 or U2-O-S/*C4orf27*$^{-/-}$ iGFP-PARP-1 cells were induced with doxycycline (+DOX) for 24 h, subjected to laser micro-irradiation and imaged by live-cell microscopy at the indicated time. Scale bar, 10 μm.

H. U2-O-S/*C4orf27*$^{+/+}$ or U2-O-S/*C4orf27*$^{-/-}$ cells were transfected with GFP-MacroD2, subjected to laser micro-irradiation and imaged by live-cell microscopy at the indicated time. Scale bar, 10 μm.

Supplemental Experimental Procedures

Lentiviral vectors and RNAi

To generate lentiviral vectors expressing GFP-tagged alleles in a doxycycline-dependent manner, full-length human C4orf27, PARP-1 and ALC1 cDNAs were each cloned into the pENTR4-GFP-C1 vector (Addgene plasmid #17396) (Campeau et al., 2009). These entry vectors were then used in an LR reaction together with the Gateway compatible pLenti-CMV/TO-Hygro destination vector (Addgene plasmid #17291), to generate pLenti-CMV/TO-Hygro-GFP-C4orf27, pLenti-CMV/TO-Hygro-GFP-PARP-1 and pLenti-CMV/TO-Hygro-GFP-ALC1. pLenti-CMV-TetR-Blast was used to generate TetR containing lentivirus (Addgene plasmid #17492). To generate lentiviral vectors expressing FLAG-tagged alleles in a constitutive manner, full length C4orf27 or the Δ3 deletion were cloned into pENTR4-FLAG (Addgene plasmid # 17423). These entry vectors were then used in an LR reaction together with the Gateway compatible pLenti-CMV-Hygro destination vector (Addgene plasmid #17454), to generate pLenti-CMV-Hygro-FLAG-C4orf27 WT or Δ3. For protein expression, C4orf27, PARP-1 and PARP-2 were cloned into the pET-28a(+) vector (Novagen) or pGEX-4T1 vector (GE Healthcare). All constructs described above were verified by Sanger sequencing. siRNAs against the C4orf27 open reading frame were purchased from Ambion (s29882 and s29883). Sequence information for each C4orf27 siRNA is available upon request. The control siRNA sequence was published previously (Gibbs-Seymour et al., 2015).

Lentivirus production

All lentiviruses were generated using the pCMV-dR8.2 dvpr and pCMV-VSV-G vectors, both kind gifts from Dr Bob Weinberg (Addgene plasmids #8455 and #8454, respectively) (Stewart et al., 2003), together with either pLenti-CMV-TetR-Blast, pLenti-CMV/TO-Hygro-GFP-C4orf27, pLenti-CMV/TO-Hygro-GFP-PARP-1 or pLenti-CMV/TO-Hygro-GFP-ALC1 vectors, as described above.

Immunochemical methods

Primary antibodies used in this study included: mouse monoclonals to ALC1 (Abcam), FLAG-M2-HRP (Sigma), γH2AX (Millipore), 6xHistidine (Clontech), Myc (Clontech), TetR (MoBiTec); rabbit polyclonals to Histone H2A (Abcam), Histone macroH2A1.1 (Abcam), Histone H3 (Millipore), Ku70 (Abcam), PARP-1 (Abcam), poly(ADP-ribose) (Trevigen), poly(ADP-ribose) (Enzo), Tubulin (Abcam), XRCC1 (Abcam); goat polyclonal to GST-HRP (Abcam). Rabbit polyclonal antibodies against C4orf27 were generated using full-length His-tagged C4orf27. Co-immunoprecipitation of protein complexes from cells followed by immunoblotting analysis was performed essentially as described previously (Gibbs-Seymour et al., 2015; Mehrotra et al., 2011). Briefly, for co-immunoprecipitation of native complexes, cells were lysed in buffer containing 50 mM Tris-HCl pH 8, 1% Triton X-100, 100 mM NaCl, Benzonase nuclease (Sigma), 1 μM PARP inhibitor (olaparib), 1 μM PARG inhibitor (ADP-HPD), 1 mM DTT, protease and phosphatase inhibitors. Lysates were clarified and added to FLAG-M2 affinity gel for 15 min whilst rotating at 4°C. Beads were washed several times with lysis buffer and eluted with LDS sample buffer (Thermo Fisher).

Mass spectrometry-based analysis of C4orf27 interactors

Protein complexes were immunopurified from 239T cells as described above. After purification, FLAG-C4orf27 or FLAG-empty vector immunoprecipitates were washed several times with lysis buffer without any Triton X-100. Beads from three independent experiments were then subjected to an on-bead tryptic digest using published FASP protocols (Wisniewski et al., 2009). Mass spectrometry was carried out essentially as described previously, using a Q Exactive mass spectrometer (Thermo, Hemel Hempstead) coupled to a Dionex Ultimate 3000 RSLCnano system (Palazzo et al., 2015). Mass spectra were searched against the Human complete proteome database using the Andromeda algorithm through MaxQuant (version 1.5.2.8) (Cox and Mann, 2008). Missing values were substituted with the minimum for each

sample and label-free quantification normalised protein intensities were log2 transformed (Cox et al., 2014). An empirical Bayes moderated t-statistics for paired samples, as implemented in the R limma package, was applied to identify proteins with a significant difference in abundance. The Benjamini and Hochberg multiple testing correction was applied to control false discovery rate.

Immunofluorescence, laser micro-irradiation and microscopy

Immunofluorescence protocols were as described previously (Gibbs-Seymour et al., 2015). Cells were pre-sensitised for 24 h before laser micro-irradiation using 10 μM BrdU (Sigma) and the media was changed to Live Cell Imaging Solution (Thermo Fisher) before laser micro-irradiation and imaging. Laser micro-irradiation was performed on an Olympus Fluoview FV1200 confocal microscope equipped an inverted IX83 motorised stage with a 37ºC humidified chamber and 60x/1.40 oil UPlanSApo objective and 405nm laser. Confocal microscopy was performed on the same microscope. Standard wide-field microscopy was performed on the Olympus BX61 microscope system, equipped with a 40x/0.75 dry objective, a CoolSNAP HQ2 camera (Roper Scientific) and MetaMorph 7.5 imaging software (GE Healthcare).

Colony formation assays

For colony formation assays, cells were plated at low densities in the presence of the indicated doses of methyl methanesulfonate (MMS). Cells were subsequently fixed and stained with crystal violet after 10 days for 293T cells. The surviving fraction at each dose was calculated after normalisation to the plating efficiency of untreated samples. For PARP inhibitor sensitivity assays, U2-O-S cells were plated in varying molarities of olaparib (stock dissolved in DMSO). Media containing fresh olaparib or vehicle was replaced every 72 h and cells were fixed and processed after 12 days as described above.

GST pull-down assays

Pull-down assays were performed essentially as described previously (Rack et al., 2015), except the TZNK/D/T buffer contained 400 mM KCl.

Circular Dichroism

Circular dichroism (CD) was carried out at 20°C using a Jasco J-815 spectropolarimeter (JASCO analytical instruments) and a 1 mm path length CD cell (Starna Scientific). Sample were purified as described above, followed by size exclusion chromatography using a HiLoad Superdex 75pg column (GE Healthcare) and 25 mM sodium phosphate pH 8 and 150 mM NaCl. This was followed by a two-step dialysis against CD buffer, 25 mM sodium phosphate pH 8 and 150 mM NaF. Samples were diluted with CD buffer to 2.5 µM before measurement. All data were background corrected and have been converted from raw ellipticity to molar residue ellipticity ($[\Theta]_{\mathrm{MRW}}$) according to Equation 1,

$$[\Theta]_{\mathrm{MRW}} = \frac{\Theta}{10 \cdot \mathrm{c_r} \cdot \ell} \tag{1}$$

where Θ is the CD signal of the sample in millidegrees; ℓ is the path length of the cell in centimetres; and c_r is the mean residue concentration in molar.

Supplemental References

Bond, C.S., and Schuttelkopf, A.W. (2009). ALINE: a WYSIWYG protein-sequence alignment editor for publication-quality alignments. Acta Crystallogr D Biol Crystallogr *65*, 510-512.

Campeau, E., Ruhl, V.E., Rodier, F., Smith, C.L., Rahmberg, B.L., Fuss, J.O., Campisi, J., Yaswen, P., Cooper, P.K., and Kaufman, P.D. (2009). A versatile viral system for expression and depletion of proteins in mammalian cells. PloS one *4*, e6529.

Cox, J., Hein, M.Y., Luber, C.A., Paron, I., Nagaraj, N., and Mann, M. (2014). Accurate proteome-wide label-free quantification by delayed normalization and maximal peptide ratio extraction, termed MaxLFQ. Mol Cell Proteomics *13*, 2513-2526.

Cox, J., and Mann, M. (2008). MaxQuant enables high peptide identification rates, individualized p.p.b.-range mass accuracies and proteome-wide protein quantification. Nat Biotechnol *26*, 1367-1372.

Gibbs-Seymour, I., Oka, Y., Rajendra, E., Weinert, B.T., Passmore, L.A., Patel, K.J., Olsen, J.V., Choudhary, C., Bekker-Jensen, S., and Mailand, N. (2015). Ubiquitin-SUMO circuitry controls activated fanconi anemia ID complex dosage in response to DNA damage. Mol Cell *57*, 150-164.

Mehrotra, P.V., Ahel, D., Ryan, D.P., Weston, R., Wiechens, N., Kraehenbuehl, R., Owen-Hughes, T., and Ahel, I. (2011). DNA repair factor APLF is a histone chaperone. Mol Cell *41*, 46-55.

Palazzo, L., Thomas, B., Jemth, A.S., Colby, T., Leidecker, O., Feijs, K.L., Zaja, R., Loseva, O., Puigvert, J.C., Matic, I., *et al.* (2015). Processing of protein ADP-ribosylation by Nudix hydrolases. Biochem J *468*, 293-301.

Rack, J.G., Morra, R., Barkauskaite, E., Kraehenbuehl, R., Ariza, A., Qu, Y., Ortmayer, M., Leidecker, O., Cameron, D.R., Matic, I., *et al.* (2015). Identification of a Class of Protein ADP-Ribosylating Sirtuins in Microbial Pathogens. Mol Cell *59*, 309-320.

Stewart, S.A., Dykxhoorn, D.M., Palliser, D., Mizuno, H., Yu, E.Y., An, D.S., Sabatini, D.M., Chen, I.S., Hahn, W.C., Sharp, P.A., *et al.* (2003). Lentivirus-delivered stable gene silencing by RNAi in primary cells. RNA *9*, 493-501.

Wisniewski, J.R., Zougman, A., Nagaraj, N., and Mann, M. (2009). Universal sample preparation method for proteome analysis. Nature methods *6*, 359-362.
