# Supplementary material for: HPF1/C4orf27 Is a PARP-1-Interacting Protein that Regulates PARP-1 ADP-Ribosylation Activity
Source: Mol Cell. 2016 May 5;62(3):432–42. doi: 10.1016/j.molcel.2016.03.008 (PMC4858568; doi:10.1016/j.molcel.2016.03.008)
Supplement: Document S2. Article plus Supplemental Information [file mmc2.pdf]

Short Article

Molecular Cell

HPF1/C4orf27 Is a PARP-1-Interacting Protein that Regulates PARP-1 ADP-Ribosylation Activity

Graphical Abstract

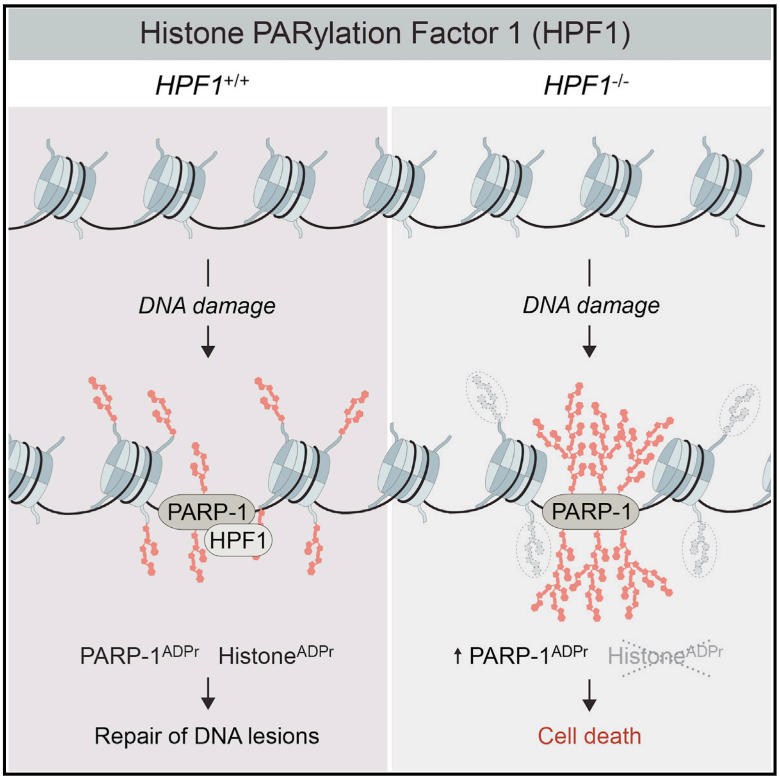

Authors

Ian Gibbs-Seymour, Pietro Fontana, Johannes Gregor Matthias Rack, Ivan Ahel

Correspondence

ivan.ahel@path.ox.ac.uk

In Brief

Gibbs-Seymour et al. identify histone PARylation factor 1 (HPF1; also known as C4orf27) as a PARP-1-interacting component of the DNA damage response. Deletion of HPF1 in human cells sensitizes cells to DNA damaging agents and to PARP inhibition. Functionally, HPF1 promotes PARP-1-dependent ADP-ribosylation of histones to ensure genome stability.

Highlights

- Histone PARylation factor 1 (HPF1) is a component of the DNA damage response
- HPF1 interacts with PARP-1 in cells via the PARP-1 catalytic domain
- Loss of HPF1 sensitizes human cells to DNA damaging agents and PARP inhibition
- HPF1 promotes PARP-1-dependent ADP-ribosylation of histones

Gibbs-Seymour et al., 2016, Molecular Cell 62, 432–442
May 5, 2016 ©2016 The Authors
http://dx.doi.org/10.1016/j.molcel.2016.03.008

Molecular Cell
Short Article

HPF1/C4orf27 Is a PARP-1-Interacting Protein that Regulates PARP-1 ADP-Ribosylation Activity

Ian Gibbs-Seymour,[1] Pietro Fontana,[1] Johannes Gregor Matthias Rack,[1] and Ivan Ahel[1,*]
[1]Sir William Dunn School of Pathology, University of Oxford, South Parks Road, Oxford OX1 3RE, UK
*Correspondence: ivan.ahel@path.ox.ac.uk
http://dx.doi.org/10.1016/j.molcel.2016.03.008

SUMMARY

We report the identification of histone PARylation factor 1 (HPF1; also known as C4orf27) as a regulator of ADP-ribosylation signaling in the DNA damage response. HPF1/C4orf27 forms a robust protein complex with PARP-1 in cells and is recruited to DNA lesions in a PARP-1-dependent manner, but independently of PARP-1 catalytic ADP-ribosylation activity. Functionally, HPF1 promotes PARP-1-dependent in *trans* ADP-ribosylation of histones and limits DNA damage-induced hyper-automodification of PARP-1. Human cells lacking HPF1 exhibit sensitivity to DNA damaging agents and PARP inhibition, thereby suggesting an important role for HPF1 in genome maintenance and regulating the efficacy of PARP inhibitors. Collectively, our results demonstrate how a fundamental step in PARP-1-dependent ADP-ribosylation signaling is regulated and suggest that HPF1 functions at the crossroads of histone ADP-ribosylation and PARP-1 automodification.

INTRODUCTION

The integrity of cellular DNA is constantly challenged by endogenous and exogenous sources of damaging agents that cause genome instability, which is a common hallmark of many different cancer types (Hanahan and Weinberg, 2011). To protect the genome from damage, organisms have evolved a cellular defense mechanism termed the DNA damage response (DDR) (Jackson and Bartek, 2009). The DDR includes a diverse set of signal transduction pathways and effector proteins that act to sense DNA lesions and effectively repair the damage to limit propagation of genomic instability to daughter cells. The complex interplay of proteins within DDR pathways requires fine spatiotemporal regulation, which is in part achieved through posttranslational modifications (PTMs), such as ADP-ribosylation, ubiquitylation, SUMOylation, phosphorylation, and acetylation. Defects in proteins involved in the DDR give rise to a range of pathologies, underlining their importance in human health and disease.

ADP-ribosylation is the enzymatic process whereby adenosine diphosphate ribose (ADPr) molecules are added to a protein, regulating various aspects of protein function, including activity or subcellular localization (Barkauskaite et al., 2015). ADP-ribosylation has a role in regulating an increasingly diverse array of cellular processes including the DDR, chromatin structure, transcriptional regulation, and RNA processing. As for many PTMs, ADP-ribosylation has "writers," "readers," and "erasers," that is, proteins which establish, recognize, or remove protein ADP-ribosylation, respectively (Barkauskaite et al., 2015). ADPr may be added as a single unit, mono(ADP-ribosyl)ation, or synthesized into polymeric chains, a process termed poly(ADP-ribosyl)ation. Mono- and poly(ADP-ribosyl)ation are catalyzed by a family of proteins called ADP-ribosyl transferases (ARTDs) or poly(ADP-ribose) polymerases (PARPs), which transfer ADPr subunits from nicotinamide adenine dinucleotide (NAD^+) to acceptor residues (Perina et al., 2014). Each PARP has distinct ADP-ribosyl transferase activities, with preferences for different substrates. In contrast to these "writers," mono(ADP-ribose) can be reversed by macrodomain-containing "eraser" proteins, such as terminal ADP-ribose glycohydrolase (TARG), while poly(ADP-ribose) can be degraded by PAR glycohydrolase (PARG) (Sharifi et al., 2013; Slade et al., 2011). Together, these "writers" and "erasers" of ADP-ribosylation ensure that this PTM is highly dynamic and fully reversible in vivo.

The founding member of the PARP family, PARP-1, has been the most widely studied of this class of enzymes. The role of PARP-1 in the DDR has been extensively described, with functions in single- and double-strand DNA break repair pathways and replication-fork-associated protection pathways. PARP-1 is activated upon binding to single- and double-strand DNA breaks via its N-terminal zinc finger domains (Ali et al., 2012; Langelier et al., 2012). Upon activation, PARP-1 extensively poly(ADP-ribosyl)ates itself, together with histones and other chromatin-associated proteins (Pascal and Ellenberger, 2015). Functionally, ADP-ribosylation of PARP-1, and presumably histones, promotes the recruitment of a range of DNA repair proteins with poly(ADP-ribose) "reader" domains that are required for lesion processing and repair. PARP-1 automodification has also been suggested to promote PARP-1 release from DNA and/or convert PARP-1 into a histone-binding protein (Muthurajan et al., 2014). Furthermore, various reports have shown that PARP-1 may be regulated by PTMs beyond its own automodification, such as phosphorylation, acetylation, SUMOylation, and ubiquitylation. However, it is not clear how these PTMs function collectively, suggesting that there might be alternative mechanisms to regulate PARP-1 ADP-ribosylation activity in vivo.

The last decade has significantly improved our understanding of the key enzymes involved in ADP-ribosylation signaling and the cellular pathways they function within. However, our understanding of how these enzymes are regulated is still in its

This is an open access article under the CC BY license (http://creativecommons.org/licenses/by/4.0/).

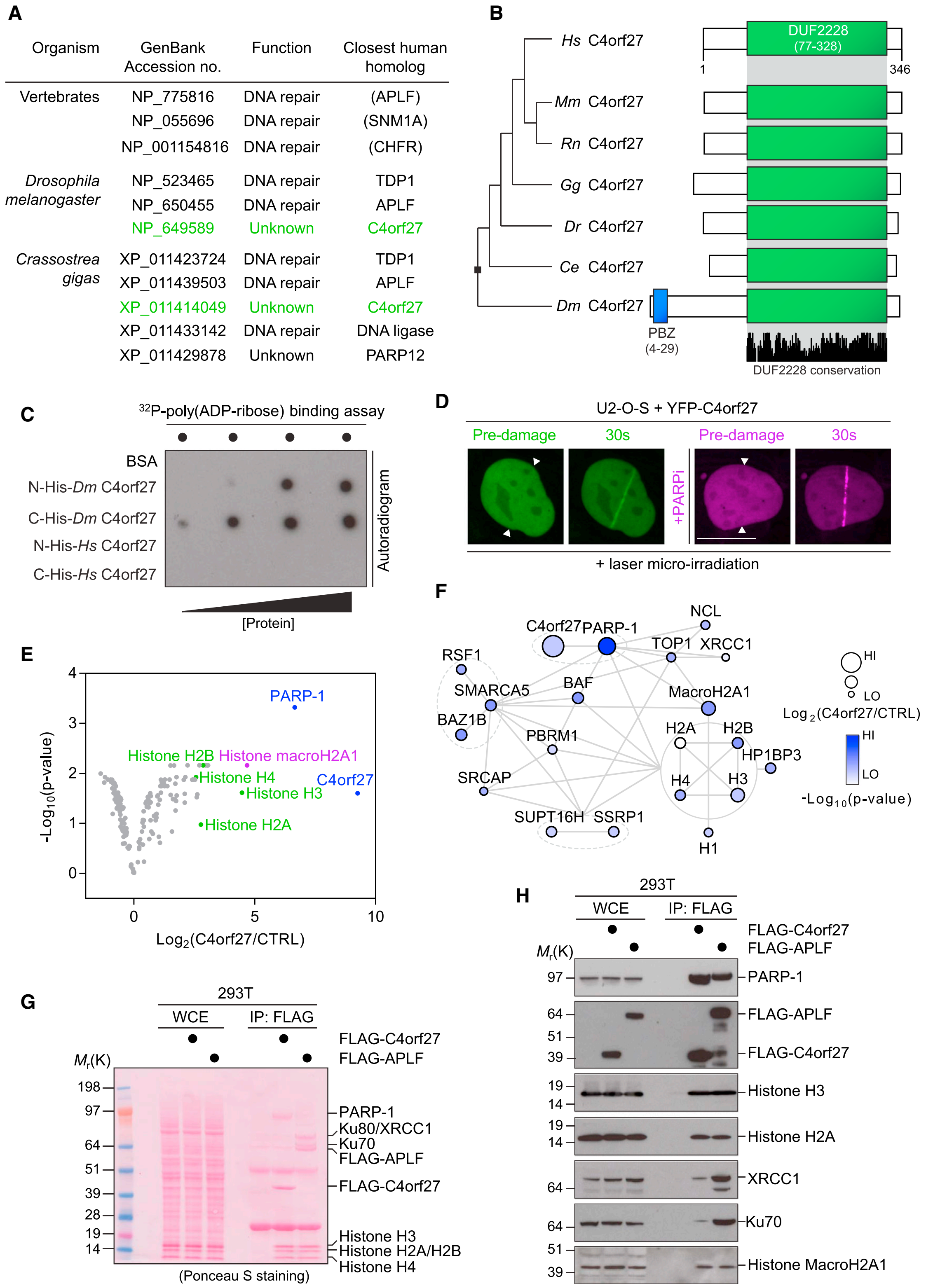

Figure 1. C4orf27/HPF1 Is a PARP-1-Interacting Factor in the DNA Damage Response

(A) Selected PBZ domain-containing proteins found in the organisms indicated in the table.

(B) Domain structure of C4orf27 from selected organisms. *Hs*, *Homo sapiens*; *Mm*, *Mus musculis*; *Rn*, *Rattus norvegicus*; *Gg*, *Gallus gallus*; *Dr*, *Danio rerio*; *Ce*, *Caenorhabditis elegans*; *Dm*, *Drosophila melanogaster*.

(legend continued on next page)

infancy. Specifically, our understanding of how PARP-1 catalytic activity is regulated in cells and how PARP-1 substrates are targeted is not well defined. Here, we identify the uncharacterized protein C4orf27 as a PARP-1-interacting factor in the DDR. C4orf27 promotes in *trans* ADP-ribosylation of histones and in turn limits DNA damage-induced hyper-automodification of PARP-1; hence, we have named the gene product HPF1, for histone PARylation factor 1. We propose that as HPF1 and PARP-1 have closely coevolved, the data presented here could therefore be a conserved mechanism of modulating PARP-1 ADP-ribosylation activity in the eukaryotic lineage.

RESULTS

C4orf27/HPF1 Is a PARP-1-Interacting Protein Recruited to DNA Lesions

Previously, we reported the identification of a poly(ADP-ribose)-binding zinc finger (PBZ) domain (Ahel et al., 2008). The PBZ domain is present in a multitude of proteins in the eukaryotic lineage (excluding yeast), and we demonstrated that it was predominantly found in proteins involved in the DDR, suggesting a close coevolution of this domain together with PARP-mediated ADP-ribosylation signaling in response to DNA lesions (Ahel et al., 2008). Since 2008, more eukaryotic genomes have been sequenced, and we now report the presence of the PBZ domain in a number of eukaryotic proteins involved in a diverse range of cellular processes, which have not previously been linked with ADP-ribosylation signaling (Figure S1A, available online). For example, these include serine/threonine kinases, ubiquitin E2 enzymes, and proteases. Though the potential human homologs of these proteins may have never acquired, or lost, a canonical PBZ domain, it suggests that novel aspects of ADP-ribosylation signaling remain to be elucidated. To probe this further, we sought to investigate one of these factors in more detail. We identified a protein in *Drosophila melanogaster* harboring a PBZ domain, termed CG1218 (Figure 1A) (Ahel et al., 2008; Isogai et al., 2010). The human homolog of CG1218 is the uncharacterized protein C4orf27. C4orf27 is highly conserved in metazoans but is also found in a small number of protozoan species. In contrast to CG1218 (referred to henceforth as *Dm* C4orf27), human C4orf27 does not contain an N-terminal PBZ domain, but instead consists largely of one domain of unknown function, DUF2228, which is common to all orthologs, as listed in the Pfam database (Figure 1B) (Finn et al., 2016). Analysis of the primary sequences from a variety of organisms revealed that only in several eukaryotic groups such as insects, sea urchins, and mollusks have C4orf27 orthologs gained a PBZ domain during evolution from the last common ancestor (Figure 1B; data not shown). To assay whether the presence of the PBZ conferred the ability to bind poly(ADP-ribose), purified recombinant C4orf27 proteins were dot-blotted onto nitrocellulose and incubated with ^{32}P-labeled poly(ADP-ribose) (Figure 1C). While human C4orf27 did not bind to poly(ADP-ribose), we observed a strong interaction of *Dm* C4orf27 with poly(ADP-ribose), consistent with the notion that the PBZ domain is the critical determinant for poly(ADP-ribose) binding in C4orf27 homologs. Given that the PBZ domain is primarily found in proteins that are involved in the DDR, we first sought to determine whether C4orf27 is a novel component of the human DDR. To this end, we generated various tools, beginning with the production of rabbit polyclonal antibodies that recognized a band at the expected molecular mass of C4orf27 (~39 kDa) by immunoblotting, which was diminished after transfection of cells with two independent siRNAs against C4orf27 (Figure S1B). Having determined the specificity of the antibody, we generated human U2-O-S and 293T *C4orf27*$^{-/-}$ cells using the CRISPR/Cas9 double nickase strategy (Figures S1C and S1D) (Ran et al., 2013). To determine if C4orf27 is actively recruited to DNA lesions, U2-O-S/*C4orf27*$^{-/-}$ cells were transfected with YFP-C4orf27 and subjected to laser microirradiation coupled to live-cell imaging. C4orf27 was recruited to DNA lesions within seconds of laser-induced damage, in a manner independent of PARP-1 catalytic activity (Figure 1D). As active generation of poly(ADP-ribose) is not the main determinant of C4orf27 localization to DNA lesions, we postulated that its recruitment might be facilitated by protein-protein interactions. Using mass spectrometry and label-free quantification of FLAG-C4orf27-associated proteins, we identified PARP-1 and the core histones (H2A, H2B, H3, and H4) among the strongest C4orf27 interactors (Figure 1E). Further hits included many proteins previously described to be regulated by PARP-1-mediated ADP-ribosylation signaling, suggesting that C4orf27 also functions within these protein networks (Figure 1F). Importantly, examination of the FLAG-C4orf27 immunoprecipitate revealed that PARP-1 was efficiently coimmunopurified with FLAG-C4orf27 (Figure 1G), which we confirmed by immunoblotting together with other hits from the C4orf27 interactome (Figure 1H). Furthermore, a phylogenetic analysis revealed that nearly all PARP-1-containing organisms have a potential C4orf27 homolog, suggesting that they might have coevolved. Collectively, our data suggest that C4orf27 forms a robust protein complex with PARP-1 in cells and is a novel factor involved in ADP-ribosylation signaling in the DDR.

C4orf27/HPF1 Chromatin Localization Is PARP-1 Dependent

To assess the specificity of the C4orf27-PARP-1 interaction, we coexpressed Myc-tagged C4orf27 together with either

(C) In vitro ^{32}P-poly(ADP-ribose) binding assay.
(D) U2-O-S cells were transfected with YFP-C4orf27, pretreated with DMSO or PARP inhibitor (PARPi), subjected to laser microirradiation, and imaged at the indicated time.
(E) Volcano plot summarizing mass spectrometry analysis of FLAG-C4orf27 interactors from n = 3 independent experiments.
(F) FLAG-C4orf27 interactors from (E) were analyzed using STRING and visualized using Cytoscape.
(G) 293T cells were transfected with FLAG-empty vector, FLAG-C4orf27, or FLAG-APLF. Cells were lysed, subjected to FLAG immunoprecipitation (IP), and analyzed by Ponceau staining of the SDS-PAGE gel. WCE, whole-cell extract.
(H) Samples shown in (G) were further analyzed by immunoblotting with the indicated antibodies.
See also Figure S1.

FLAG-tagged PARP-1, PARP-2, or PARP-3 in 293T/*C4orf27*$^{-/-}$ cells. As expected, we observed a strong interaction between C4orf27 and PARP-1; however, we also observed a weak but reproducible interaction between C4orf27 and PARP-2, suggesting that although C4orf27 primarily binds PARP-1, it does have the ability to interact with PARP-2, at least under these overexpressed conditions (Figure 2A). As the C4orf27-PARP-1 complex coimmunopurifies all core histones (Figure 1), we used 293T/*PARP-1*$^{-/-}$ cells (Figure S1D) to determine whether the C4orf27-histone interaction is mediated by PARP-1. Indeed, coimmunopurification followed by immunoblotting revealed that PARP-1 mediates the interaction between C4orf27 and histones, as loss of PARP-1 abrogated the ability of C4orf27 to interact with histones in vivo (Figure 2B). Based on the above data and our previous observation that C4orf27 recruitment to DNA lesions is independent of PARP-1 catalytic activity, we examined whether C4orf27 recruitment to DNA lesions was dependent on the interaction with PARP-1. To achieve this, we generated a cellular system whereby doxycycline addition led to the inducible expression of GFP-tagged C4orf27 (iGFP-C4orf27) in various U2-O-S genetic backgrounds (Figure S2A). Using these cells, we observed that loss of PARP-1 inhibited C4orf27 recruitment to DNA lesions, suggesting that a functional complex between PARP-1 and C4orf27 is essential to promote C4orf27 localization at DNA lesions (Figures 2C and 2D). The poly(ADP-ribose)-binding protein ALC1 served as a control in this assay (Figures S2C and S2D). We then revisited our previous observation that pretreatment of cells with PARP inhibitor (PARPi) did not abolish C4orf27 recruitment to sites of DNA damage. Notably, pretreatment of cells with PARPi resulted in a stronger initial recruitment of C4orf27 and pronounced delay in the removal of the C4orf27-PARP-1 complex at later time points (Figure 2E). As a control for the efficacy of PARPi treatment in this assay, ALC1 recruitment to DNA lesions was sensitive to PARP inhibition (Figure S2E), as previously shown (Ahel et al., 2009). In contrast, PARP-1 was trapped at DNA lesions when cells were pretreated with PARPi, in the same manner as C4orf27 (Figures S2B, S2F, and S2G). Thus, given that C4orf27 recruitment to DNA lesions is PARP-1 dependent, these data suggest that PARP inhibition cotraps the C4orf27-PARP-1 complex at sites of DNA damage. Taken together, our data suggest that C4orf27 requires the physical interaction with PARP-1, but not its catalytic activity, for localization to DNA lesions and chromatin.

The C4orf27/HPF1 C-Terminal Region Is Important for PARP-1 Interaction

The above data indicated that the interaction between C4orf27 and PARP-1 might be independent of active PARP-1 ADP-ribosylation. To test this further, cells were treated with PARPi in vivo before analysis of protein complex formation by coimmunopurification and immunoblotting. While the interaction between APLF and PARP-1 was PARPi sensitive, as we previously reported, the interaction between C4orf27 and PARP-1 was unaffected by PARPi treatment (Figure 3A). Furthermore, the interaction between C4orf27 and PARP-1 was unchanged after DNA damage, consistent with the notion that the two form a protein complex that is recruited to DNA lesions via PARP-1 (Figure 3B).

We then sought to define the minimal region of C4orf27 necessary for PARP-1 binding. Using a series of C4orf27 deletion constructs based on secondary structure predictions (Figure S3A), we found that the C-terminal region encompassing amino acids 242–346 of C4orf27 was important for the interaction with PARP-1 (Figure 3C). Consistent with our previous data, the abrogation of PARP-1 binding in the C4orf27 Δ3 mutant inhibited the ability of C4orf27 to coimmunopurify histones. Furthermore, the C4orf27 Δ3 mutant, despite localizing to the nucleus, was defective for recruitment to sites of laser-induced DNA damage, again underlining the importance of a functional C4orf27-PARP-1 interaction for C4orf27 recruitment to DNA lesions (Figures 3D, S3B, and S3C). Moreover, in a search for key residues in the C-terminal region, we mutated two highly conserved amino acids, Tyr238 and Arg239, which are both found in all C4orf27 orthologs, either singly or doubly, to alanine (Figure S3A). Despite being exclusively nuclear, all three of these mutants were unable to bind PARP-1 and histones, suggesting that these residues are important for the C4orf27-PARP-1 interaction (Figure S3B).

Based on our in vivo interaction and recruitment data, we hypothesized that the interaction between C4orf27 and PARP-1 might be direct. Moreover, the observation of an interaction between C4orf27 and PARP-2 was suggestive of a common interface on PARP-1/PARP-2 that interacts with C4orf27, possibly mediated by the C-terminal region of PARP-1/PARP-2, which they share. Indeed, GST pull-down assays with recombinant PARP-1 fragments revealed that the PARP-1 catalytic (CAT) domain interacted directly with recombinant C4orf27 (Figure 3E). Furthermore, recombinant PARP-2 CAT domain was also able to pull down C4orf27 in our in vitro conditions (Figure 3F). Taken together with our in vivo data, our results reveal that C4orf27 is localized to sites of DNA damage via a direct interaction between C4orf27 and the PARP CAT domain.

C4orf27/HPF1 Promotes Histone ADP-Ribosylation and Limits Hyper-automodification of PARP-1

The role of PARP-1 in the DDR as a genome caretaker is well established. At the cellular level, *Parp-1*$^{-/-}$ mouse embryonic fibroblasts are sensitive to a range of genotoxic agents, particularly those that cause single-strand DNA breaks (El-Khamisy et al., 2003). Therefore, we analyzed the sensitivity of human cells null for *C4orf27*, *PARP-1*, or *C4orf27/PARP-1* to MMS, a potent inducer of single-strand DNA breaks. We observed that 293T/*C4orf27*$^{-/-}$ cells were extremely sensitive to MMS, though slightly less so than *PARP-1*$^{-/-}$ cells (Figure 4A). Notably, *C4orf27*$^{-/-}$*PARP-1*$^{-/-}$ appeared to have a modest additive impact on cellular sensitivity to MMS compared to single knockouts, suggesting that C4orf27 may have DNA repair functions beyond the C4orf27-PARP-1 complex (Figure 4A). As we demonstrated above, C4orf27 is cotrapped with PARP-1 at DNA lesions if cells are pretreated with PARPi before DNA damage induction. Therefore, we considered how C4orf27 loss might impact cellular fitness in the presence of PARPi by analyzing long-term colony formation. Strikingly, loss of C4orf27 sensitized cells to PARPi, a phenotype that could be fully rescued in the *C4orf27*$^{-/-}$*PARP-1*$^{-/-}$ background (Figure 4B). Collectively, our data demonstrate that C4orf27 acts with PARP-1 to promote genome stability in response to DNA damage and further

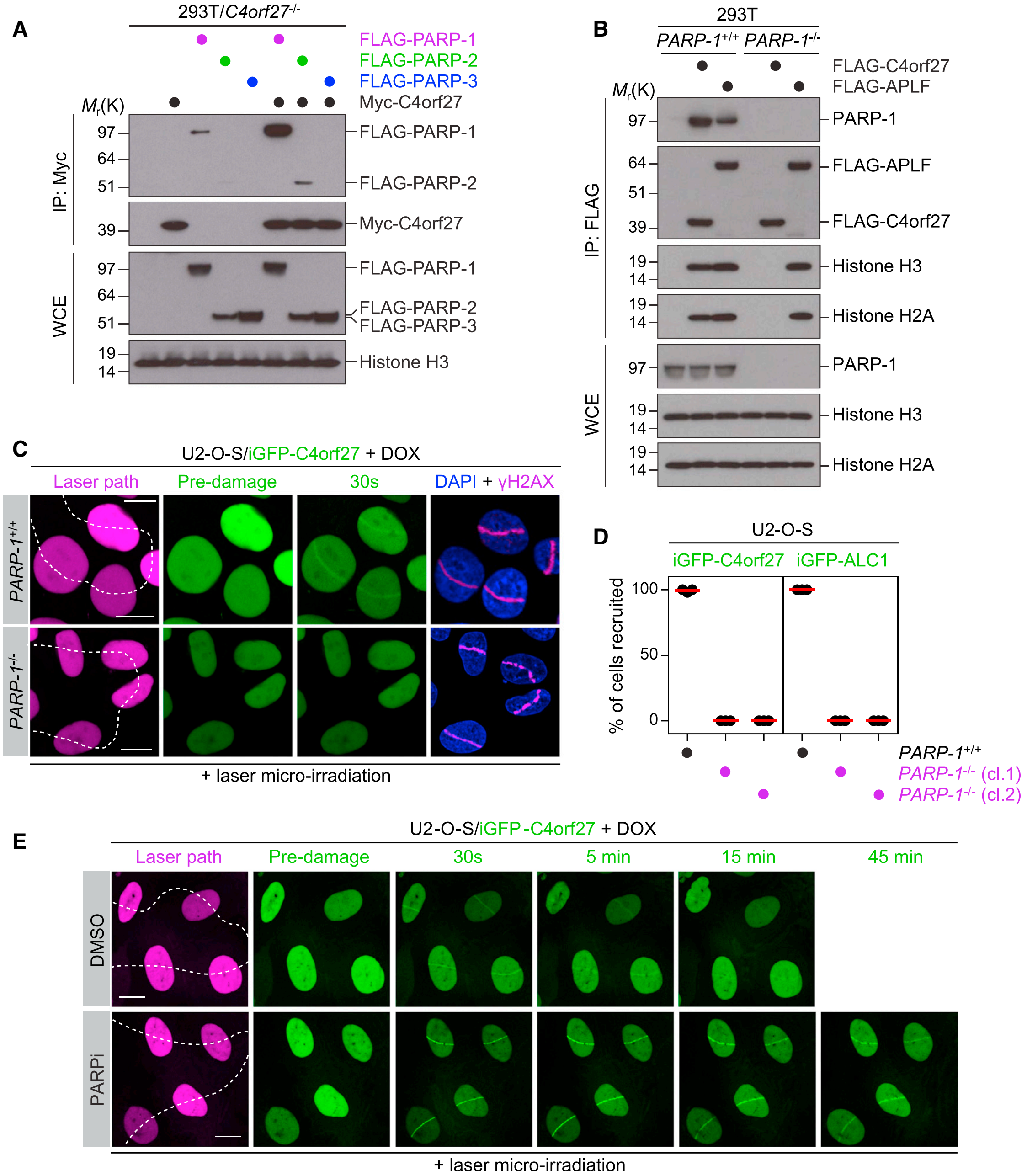

Figure 2. C4orf27/HPF1 Recruitment to Chromatin Is PARP-1 Dependent but Independent of PARP-1 Catalytic Activity

(A) 293T/*C4orf27*$^{-/-}$ cells were transfected with the indicated combinations of FLAG-empty vector, FLAG-PARP-1, FLAG-PARP2, or FLAG-PARP3 together with either Myc-empty vector or Myc-C4orf27, purified with Myc-tag agarose, and analyzed by immunoblotting with the indicated antibodies.

(B) 293T or 293T/*PARP-1*$^{-/-}$ cells were transfected with FLAG-empty vector, FLAG-C4orf27, or FLAG-APLF, subjected to FLAG immunoprecipitation, and analyzed by immunoblotting with the indicated antibodies.

(C) U2OS/*PARP-1*$^{+/+}$ iGFP-C4orf27 or U2OS/*PARP-1*$^{-/-}$ iGFP-C4orf27 cells were induced with doxycycline (+DOX) for 24 hr, subjected to laser microirradiation, and imaged by live-cell microscopy at the indicated time. Cells were subsequently fixed and immunostained with γH2AX antibody. Scale bar, 10 μm.

(D) Quantification of data shown in (C). Data represent mean ± SEM from three biologically independent experiments. At least 50 cells were quantified in each experiment.

(E) U2OS/iGFP-C4orf27 cells were induced with DOX for 24 hr, pretreated with DMSO or PARPi for 1 hr, and then subjected to laser microirradiation and imaged by live-cell microscopy at the indicated time. Scale bar, 10 μm.

See also Figure S2.

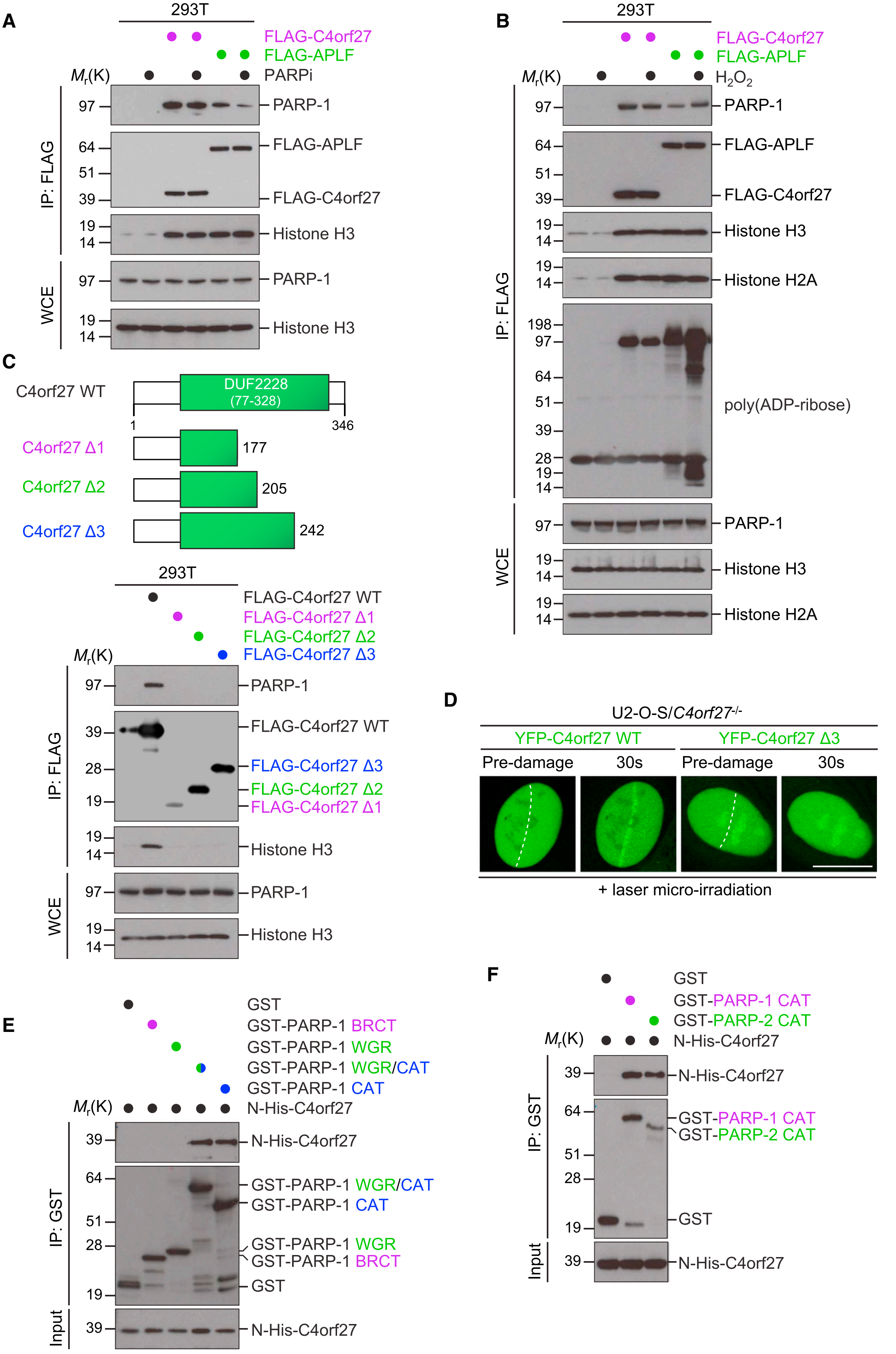

(legend on next page)

suggest that C4orf27 is an important modulator of PARPi-mediated toxicity in cells.

Despite extensive bioinformatics effort, we were unable to predict a function for C4orf27 based on the primary sequence. However, given the strong interaction between C4orf27 and PARP-1 in cells, the close coevolution of the two genes, and the phenotypes described above, we reasoned that C4orf27 might have an intrinsic role in PARP-1 catalytic activity. Therefore, we analyzed the products of PARP-1-mediated ADP-ribosylation activity after DNA damage. The two major targets of PARP-1 catalytic activity are PARP-1 itself and histones, which are both clearly evident by immunoblotting of whole-cell extracts with anti-poly(ADP-ribose) antibodies. After DNA damage, we observed PARP-1 hyper-automodification in U2-O-S/*C4orf27*$^{-/-}$ cells, with a concomitant loss of histone ADP-ribosylation in these cells (Figure 4C). These results were confirmed by western blotting of FLAG-histone H2B immunoprecipitates with anti-poly(ADP-ribose) antibodies, underlining a key role for C4orf27 in promoting histone ADP-ribosylation and limiting PARP-1 hyper-automodification (Figure S4A). We then complemented U2-O-S/*C4orf27*$^{+/+}$ and U2-O-S/*C4orf27*$^{-/-}$ cells with either empty vector, FLAG-C4orf27 WT (wild-type), or FLAG-C4orf27 Δ3. Interestingly, overexpression of FLAG-C4orf27 WT in U2-O-S/*C4orf27*$^{+/+}$ caused sensitivity to PARPi, while the FLAG-C4orf27 Δ3 mutant rescued this phenotype, suggesting the interaction with PARP-1 mediated this sensitivity in a WT genetic background (Figure S4B). Thus, the dominant-negative impact of overexpression of FLAG-C4orf27 WT caused only minor rescue of the sensitivity of U2-O-S/*C4orf27*$^{-/-}$ cells to PARPi in clonogenic assays (Figure S4B). Western blot analysis revealed that overexpression of FLAG-C4orf27 WT, but not the Δ3 mutant, inhibited PARP-1 activity after DNA damage, limiting PARP-1 automodification and only partially rescuing histone ADP-ribosylation (Figure S4C). Therefore, while an absence of C4orf27 prevents PARP-1 hyper-automodification and promotes histone ADP-ribosylation, overexpression of C4orf27 inhibits PARP-1-dependent ADP-ribosylation signaling.

To examine our in vivo observations further, we reconstituted the PARP-1-dependent ADP-ribosylation reaction in vitro, using recombinant PARP-1 and C4orf27 together with NAD^+ and DNA to activate the reaction. The addition of WT C4orf27, but not the Y238A/R239A mutant, to the in vitro reaction produced a robust reduction of PARP-1 automodification (Figure 4D), which was not due to C4orf27 poly(ADP-ribose) glycohydrolase activity (Figure S4D). Given that the C4orf27-PARP-1 complex also coimmunopurified histones from cells, we examined how histones might impact the reaction. Importantly, when we added recombinant histone octamer to the in vitro ADP-ribosylation reaction, C4orf27 was able to promote PARP-1-mediated ADP-ribosylation of the histone octamer, but not APLF, another PARP-1 target (Figure 4E). We also observed this activity for histone H1 (Figure 4F) and the histone H3/H4 tetramer (Figure S4E). To exclude the possibility that misfolding of the mutant C4orf27 was responsible for these observations, we performed circular dichroism analysis and did not detect any notable differences between WT C4orf27 and the Y238A/R239A mutant (Figure S4F). Collectively, the in vitro data corroborate our in vivo findings, suggesting that C4orf27 acts to promote PARP-1-dependent in *trans* ADP-ribosylation of histones and limit PARP-1 hyper-automodification.

To translate these findings back into a cellular context, we analyzed how C4orf27 loss impacted ADP-ribosylation signaling at DNA lesions. Loss of C4orf27 did not impact PARP-1 association with histones in undamaged conditions (data not shown), nor did it impact PARP-1 recruitment to DNA lesions (Figure S4G). However, we observed that PARP-1 was retained longer at DNA lesions in U2-O-S/*C4orf27*$^{-/-}$ cells compared to WT cells (Figure S4G). Based on these findings and our previous observations, we hypothesized that readers of PARP-1 ADP-ribosylation signaling might exhibit altered recruitment dynamics to DNA lesions in *C4orf27*$^{-/-}$ cells if the reader binds preferentially to automodified PARP-1 or ADP-ribosylated histones. Previous reports have shown that ALC1 is a reader of PARP-1-dependent ADP-ribosylation signaling and possibly automodified PARP-1 itself (Ahel et al., 2009; Gottschalk et al., 2012). We found that ALC1 displayed prolonged retention at DNA lesions in the absence of C4orf27, correlating with our observations for PARP-1, implying that the hyper-automodified form of PARP-1 that occurs in the absence of C4orf27 promotes extended interaction with ALC1 at DNA lesions (Figure 4G). Conversely, we reasoned that proteins that interact with hypo-ADP-ribosylated PARP-1 might exhibit reduced recruitment to sites of DNA damage in *C4orf27*$^{-/-}$ cells, where the hypermodified form of PARP-1 persists. Indeed, analysis of one such protein, MacroD2, revealed markedly reduced recruitment to sites of DNA damage in *C4orf27*$^{-/-}$ cells (Figure S4H). Collectively, our converging lines of in vivo and in vitro evidence suggest that C4orf27 is a key regulator of PARP-1-dependent ADP-ribosylation signaling, acting to promote histone ADP-ribosylation, limit DNA damage-induced PARP-1 hyper-automodification, and ensure genome stability.

Figure 3. C4orf27/HPF1 Interacts Directly with PARP-1

(A) 293T cells were transfected with FLAG-empty vector, FLAG-C4orf27, or FLAG-APLF and treated with PARPi or vehicle. Cells were lysed, subjected to FLAG immunoprecipitation, and analyzed by immunoblotting with the indicated antibodies.

(B) As for (A) except cells were treated with either vehicle or H_2O_2 (1 μM) for 10 min.

(C) 293T/*C4orf27*$^{-/-}$ cells were transfected with FLAG-empty vector, FLAG-C4orf27 WT, or deletion mutants Δ1, Δ2, or Δ3, subjected to FLAG immunoprecipitation, and analyzed by immunoblotting with the indicated antibodies.

(D) U2-O-S/*C4orf27*$^{-/-}$ cells were transfected with YFP-C4orf27 WT or Δ3, subjected to laser microirradiation, and imaged by live-cell microscopy at the indicated time. Scale bar, 10 μm.

(E) Recombinant His-C4orf27 was incubated with GST alone or with various GST-PARP-1 fragments. Samples were subjected to GST pull-down, and bound complexes were analyzed by immunoblotting with the indicated antibodies.

(F) As in (E) except with either GST-PARP-1 CAT domain or GST-PARP-2 CAT domain.

See also Figure S3.

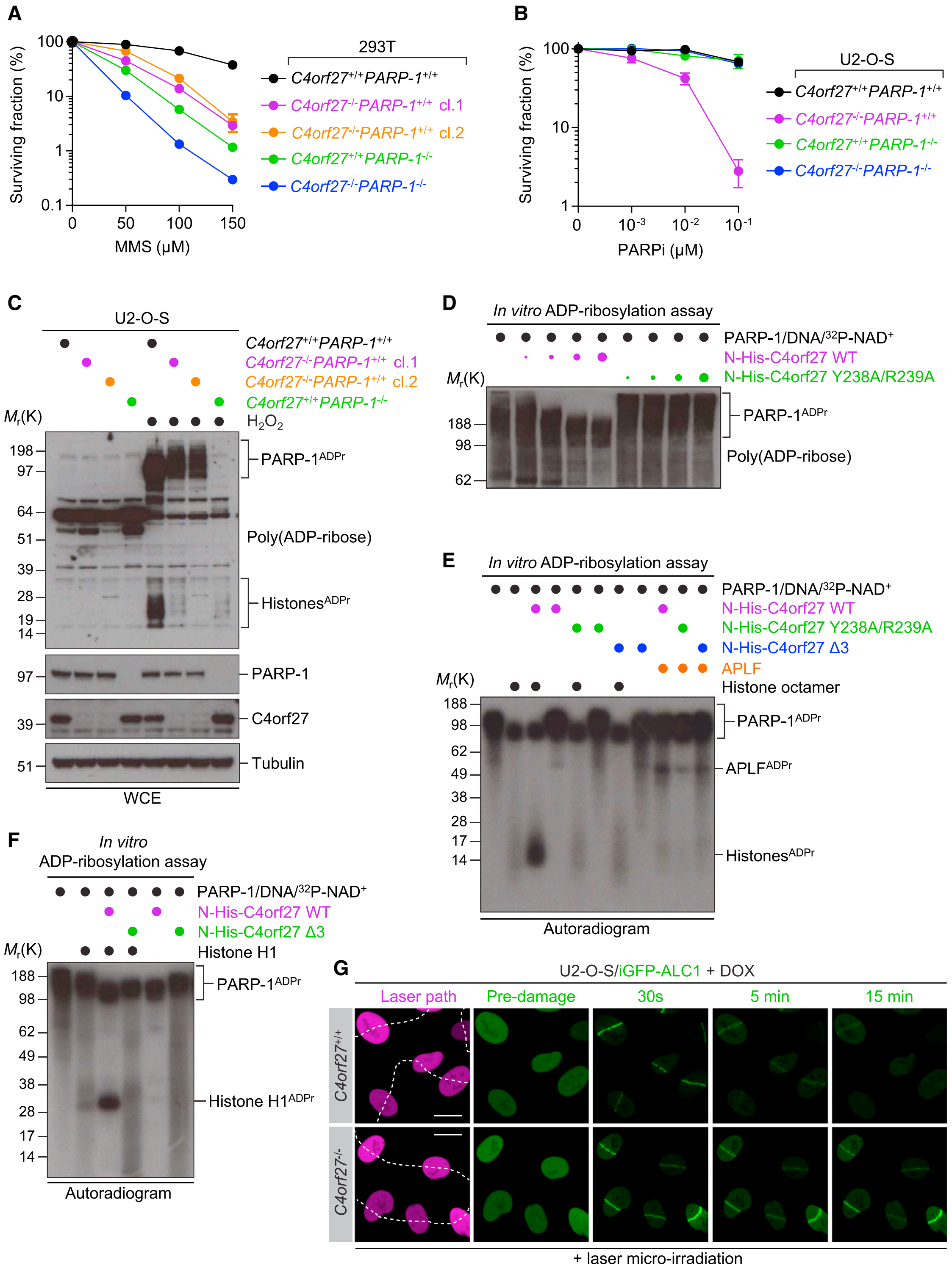

Figure 4. C4orf27/HPF1 Suppresses PARP-1 Hyper-automodification and Promotes Histone ADP-Ribosylation

(A) Clonogenic survival of indicated 293T cell lines treated with various doses of methyl methanesulfonate (MMS). Data represent mean ± SEM from two biologically independent experiments using technical triplicates per data point.

(B) Clonogenic survival of indicated U2-O-S cell lines treated with various doses of PARPi. Data represent mean ± SEM from three biologically independent experiments using technical triplicates per data point.

(legend continued on next page)

DISCUSSION

Our understanding of the PARP-1 structure and catalytic mechanism has been significantly advanced by recent progress in the field (Barkauskaite et al., 2015; Pascal and Ellenberger, 2015). However, how PARP-1 activity is regulated in vivo and how PARP-1 achieves substrate selectivity is poorly understood. Here, we have identified the uncharacterized protein C4orf27 as a PARP-1-interacting factor in the DNA damage response. C4orf27 exhibits a robust interaction with PARP-1 in cells, suggesting that the interaction is functionally important. Indeed, we have provided several lines of evidence that C4orf27 acts to promote in *trans* ADP-ribosylation of histones, and as such, we have named the protein HPF1, for histone PARylation factor 1. In the absence of HPF1, PARP-1 is unable to ADP-ribosylate its main substrates, histones, and as a result undergoes DNA damage-induced hyper-automodification. We propose that as HPF1 and PARP-1 have closely coevolved, the data presented here could therefore be a conserved mechanism of modulating PARP-1 ADP-ribosylation activity in the eukaryotic lineage. Furthermore, although we have shown that HPF1 can modulate PARP-1 in the context of the DNA damage response, we fully expect that multiple facets of PARP-1 function may be regulated by the interaction with HPF1. It will be important to broaden our understanding of HPF1 function in various other PARP-1-dependent ADP-ribosylation processes in the future, both at the cellular and organismal level.

A quantification of protein concentrations in HeLa cells revealed that PARP-1 far exceeds that of HPF1 (~2,030 nM versus ~104 nM), suggesting the existence of multiple PARP-1 protein complexes in addition to the HPF1-PARP-1 complex (Hein et al., 2015). Supporting this, a robust PARP-1-Timeless protein complex interaction was recently identified (Xie et al., 2015; Young et al., 2015). Similar to Timeless, we observed that HPF1 is co-trapped with PARP-1 at DNA lesions upon PARPi treatment. However, we did not identify any Timeless peptides in our unbiased mass spectrometry analysis of HPF1-interacting proteins, suggesting that the HPF1-PARP-1 and Timeless-PARP-1 complexes are distinct. Moreover, Timeless had no impact on PARP-1 ADP-ribosylation activity in vitro (Xie et al., 2015). This is in stark contrast to HPF1, suggesting further separation of function between these two protein complexes. Xie et al. also provided the structural basis for the PARP-1-Timless interaction, which is through the PARP-1 catalytic domain. Similarly, here we have provided evidence that the HPF1-PARP-1 interaction is also via the PARP-1 catalytic domain. Thus, in the future it will be essential to determine the structural relationship between HPF1 and PARP-1 to define further how the interaction is able to modulate PARP-1-dependent ADP-ribosylation of histones.

Treatment of cells with PARPi causes trapping of PARP-1 on DNA, which both prevents the subsequent recruitment of DNA repair proteins (through catalytic inhibition) and may also eventually lead to the formation of double-strand break when a replication fork meets the DNA-trapped PARP-1 (Helleday, 2011). This collapsed replication fork may then become a substrate for the homologous recombination (HR) machinery, which is why HR-deficient cells might be so exquisitely sensitive to PARPi. Interestingly, our finding that the loss of HPF1 is chemically lethal with PARPi raises a number of important questions. For example, is the HPF1-PARP-1 complex also required to promote homologous recombination or inhibit nonhomologous end joining at stalled or collapsed replication forks? Or does a lack of HPF1 result in more DNA-trapped PARP-1?

In summary, we identify the previously uncharacterized protein HPF1 as a PARP-1-interacting factor in the DDR. We demonstrate that HPF1 acts to modulate PARP-1-dependent ADP-ribosylation signaling, promoting in *trans* ADP-ribosylation of histones and thereby suppressing DNA damage-induced PARP-1 hyper-automodification. We speculate that the close coevolution of the two proteins represents a well-conserved mechanism to regulate this fundamental step in ADP-ribosylation signaling.

EXPERIMENTAL PROCEDURES

Plasmids and CRISPR/Cas9 sgRNA Sequences

Full-length human C4orf27 cDNA was purchased and cloned into the pDONR221 vector (Thermo Fisher). Mammalian expression constructs were generated using pDONR221-C4orf27 and appropriate destination vectors using the LR Clonase II enzyme mix (Thermo Fisher) to generate N-terminal YFP-, Myc-, or FLAG-tagged C4orf27. Point mutants and deletions were produced in pDONR221-C4orf27 using Phusion High-Fidelity DNA Polymerase (NEB). Full-length PARP-1, PARP-2, and PARP-3 cDNAs were cloned into pDONR221 before generating FLAG-PARP-1, FLAG-PARP-2, and FLAG-PARP-3. GFP-MacroD2 was a kind gift from Gyula Timinszky (Timinszky et al., 2009). FLAG-APLF was described previously (Mehrotra et al., 2011). The pSpCas9n(BB)-2A-Puro (PX462) V2.0 vector was a kind gift from Dr. Feng Zhang (Addgene plasmid #62987) (Ran et al., 2013). The following single guide RNA (sgRNA) sequences were used in this study: for C4orf27-sgRNA#1, 5′-CAGCAGAATTCCCCGATCCG- 3′ (exon 1); for C4orf27-sgRNA#2, 5′-TCG GCGGTGGCGGGAAGCGC- 3′ (exon 1); for PARP-1-sgRNA#1, 5′-CCACCT CAACGTCAGGGTGC- 3′ (exon 2); and for PARP-1-sgRNA#2, 5′-TGGGTT CTCTGAGCTTCGGT- 3′ (exon 2).

Cell Culture

Human U2-O-S and HEK293T cells (abbreviated to 293T) were cultured in DMEM (Sigma) containing 10% FBS and penicillin/streptomycin (both Thermo

(C) U2-O-S cells with the indicated genetic backgrounds were treated with H_2O_2 (1 μM) for 10 min, and whole-cell extracts were analyzed by immunoblotting with the indicated antibodies. ADP-ribosylated PARP-1 (PARP-1^{ADPr}) and histones ($Histones^{ADPr}$) are indicated.
(D) Recombinant PARP-1 and increasing molarities of recombinant N-His-C4orf27 were incubated with NAD^+ and DNA. Reaction products were separated by SDS-PAGE and analyzed by immunoblotting with anti-poly(ADP-ribose) antibody.
(E) Recombinant PARP-1, N-His-C4orf27 WT, N-His-C4orf27 Y238A/R239A, N-His-C4orf27 Δ3, or APLF was incubated in various combinations with recombinant histone octamer, and reactions were initiated by addition of DNA and ^{32}P-NAD^+. Reaction products were analyzed by SDS-PAGE and autoradiography.
(F) Similar to (E), except recombinant histone H1 was used.
(G) U2-O-S/*C4orf27*$^{+/+}$ iGFP-ALC1 or U2-O-S/*C4orf27*$^{-/-}$ iGFP-ALC1 cells were induced with DOX for 24 hr, subjected to laser microirradiation, and imaged at the indicated times. Scale bar, 10 μm.
See also Figure S4.

Fisher). Transient DNA transfections were performed with TransIT-LT1 (Mirus), Fugene6 (Promega), or Polyfect (QIAGEN), and transient small interfering RNA (siRNA) transfections were performed with Lipofectamine RNAiMAX (Thermo Fisher), each according to the manufacturer's instructions. To generate CRISPR/Cas9-mediated knockout cell lines, U2-O-S and 293T were transfected with pairs of the PX462 vector containing either C4orf27-sgRNA#1 or C4orf27-sgRNA#2 for *C4orf27* knockouts and either PARP-1-sgRNA#1 or PARP-1-sgRNA#2 for *PARP-1* knockouts. All four vectors were used together for double *C4orf27/PARP-1* knockouts. Transfected cells were plated at low density in 1 μg/ml puromycin (Invivogen). Single colonies were propagated, and individual clones were assessed by western blotting. To generate doxycycline-inducible cell lines, U2-O-S/$C4orf27^{+/+}PARP\text{-}1^{+/+}$, U2-O-S/$C4orf27^{-/-}PARP\text{-}1^{+/+}$, and U2-O-S/$C4orf27^{+/+}PARP\text{-}1^{-/-}$ cells were first transduced with lentivirus generated from pLenti-CMV-TetR-Blast and selected in 15 μg/ml blasticidin (Invivogen). Stable cells were subsequently transduced with lentivirus generated from pLenti-CMV/TO-Hygro-GFP-C4orf27, pLenti-CMV/TO-Hygro-GFP-PARP-1, or pLenti-CMV/TO-Hygro-GFP-ALC1 constructs and selected in 500 μg/ml hygromycin B (Thermo Fisher). Doxycycline hyclate (Sigma) was added to media at a final concentration of 1 μg/ml for 24 hr. For cellular H_2O_2 (Sigma) treatments, the H_2O_2 stock (~8.8 M) was diluted in PBS containing $MgCl_2$ and $CaCl_2$ and added to cells for the time indicated in the text. For PARP inhibitor treatments, cells were treated with 1 μM olaparib for 12 hr for coimmunoprecipitation experiments or with 10 μM olaparib for 1 hr for laser microirradiation experiments.

Recombinant Protein Production

Recombinant PARP-1 was from Trevigen (high specific activity), histone H1 and histone H3/H4 tetramer were from NEB, and histone octamer was prepared as described previously (Ahel et al., 2009; Mehrotra et al., 2011). All recombinant proteins were expressed in Rosetta (DE3) cells (Novagen), and isolated single colonies were grown in LB medium. Expression was induced at OD_{600} 0.6 with 1 mM IPTG overnight at 18°C. His-tagged proteins were purified by Ni^{2+}-NTA chromatography (QIAGEN) according to the manufacturer's protocol using the following buffer: 50 mM Tris-HCl (pH 8.0) and 500 mM NaCl supplemented with protease inhibitors and Benzonase nuclease (Novagen). Imidazole molarities used in the buffer were as follows: lysis, 20 mM; wash, 40 mM; elution, 500 mM. Proteins were dialysed in 50 mM Tris-HCl (pH 8.0), 100 mM NaCl, and 1 mM DTT. GST-tagged proteins were purified using Glutathione Sepharose 4B (GE Healthcare) according to the manufacturer's protocol using PBS containing 1 mM DTT supplemented with protease inhibitors and Benzonase nuclease. Proteins were eluted with 50 mM Tris-HCl (pH 80, 200 mM NaCl, 1 mM DTT, and 10 mM reduced glutathione. Proteins were dialyzed overnight against 50 mM Tris-HCl (pH 8), 150 mM KCl, 12 mM NaCl, 2 mM $MgCl_2$, 5 mM DTT, and 0.1% Triton X-100.

Immunofluorescence, Laser Microirradiation, and Microscopy

Protocols were as previously described (Gibbs-Seymour et al., 2015).

In Vitro ADP-Ribosylation Assays

^{32}P-poly(ADP-ribose) binding assays were performed as described previously (Ahel et al., 2008). In vitro ADP-ribosylation assays performed essentially as described previously (Ahel et al., 2008; Barkauskaite et al., 2013). Recombinant proteins were mixed before the addition of NAD^+ to the reaction, which proceeded for 20 min. PARP inhibitor (olaparib) was added at the end of the reactions before subsequent analysis by immunoblotting or autoradiography. The molarity of PARP-1 used in the reaction was 0.1 μM, histones were 1 μM, and C4orf27 ranged from 1 to 25 μM. For poly(ADP-ribose) glycohydrolase activity assay, PARP-1 was first automodified by addition of ^{32}P-NAD^+ and DNA for 20 min, and the reaction was stopped by olaparib addition. Recombinant PARG or C4orf27 was then added to the reaction for a further 30 min, and products were analyzed by autoradiography.

SUPPLEMENTAL INFORMATION

Supplemental Information includes Supplemental Experimental Procedures and four figures and can be found with this article online at http://dx.doi.org/10.1016/j.molcel.2016.03.008.

AUTHOR CONTRIBUTIONS

I.G.-S. designed and performed experiments, analyzed data, and cowrote the manuscript; P.F. purified recombinant proteins and performed biochemical and biophysical assays; J.G.M.R. purified recombinant proteins and performed CD analysis; and I.A. supervised the project, designed experiments, analyzed data, and cowrote the manuscript.

ACKNOWLEDGMENTS

We thank Drs. Bob Weinberg, Feng Zhang, Gyula Timinszky, and Eric Campeau for the invaluable provision of reagents, and Dr. Ben Thomas, Dr. Elisa Pappalardo, and Svenja Hester (Sir William Dunn School of Pathology, University of Oxford) for help with mass spectrometry and analysis. We also thank Drs. Anja Groth and Martina Hödl-Baumgartner (Biotech Research and Innovation Centre, University of Copenhagen) and Drs. Grigory Dianov and Mattia Poletto (Department of Oncology, University of Oxford) for sharing unpublished results. We also thank Gytis Jankevicius (Sir William Dunn School of Pathology) for critical reading of the manuscript. This work was supported by grants from the Wellcome Trust (grant number 101794) and European Research Council (grant number 281739) (both to I.A.). I.G.-S. is a Marie Skłodowska-Curie Fellow.

Received: November 2, 2015
Revised: February 2, 2016
Accepted: March 4, 2016
Published: April 7, 2016

REFERENCES

Ahel, I., Ahel, D., Matsusaka, T., Clark, A.J., Pines, J., Boulton, S.J., and West, S.C. (2008). Poly(ADP-ribose)-binding zinc finger motifs in DNA repair/checkpoint proteins. Nature *451*, 81–85.

Ahel, D., Horejsí, Z., Wiechens, N., Polo, S.E., Garcia-Wilson, E., Ahel, I., Flynn, H., Skehel, M., West, S.C., Jackson, S.P., et al. (2009). Poly(ADP-ribose)-dependent regulation of DNA repair by the chromatin remodeling enzyme ALC1. Science *325*, 1240–1243.

Ali, A.A., Timinszky, G., Arribas-Bosacoma, R., Kozlowski, M., Hassa, P.O., Hassler, M., Ladurner, A.G., Pearl, L.H., and Oliver, A.W. (2012). The zinc-finger domains of PARP1 cooperate to recognize DNA strand breaks. Nat. Struct. Mol. Biol. *19*, 685–692.

Barkauskaite, E., Brassington, A., Tan, E.S., Warwicker, J., Dunstan, M.S., Banos, B., Lafite, P., Ahel, M., Mitchison, T.J., Ahel, I., and Leys, D. (2013). Visualization of poly(ADP-ribose) bound to PARG reveals inherent balance between exo- and endo-glycohydrolase activities. Nat. Commun. *4*, 2164.

Barkauskaite, E., Jankevicius, G., and Ahel, I. (2015). Structures and mechanisms of enzymes employed in the synthesis and degradation of PARP-dependent protein ADP-ribosylation. Mol. Cell *58*, 935–946.

El-Khamisy, S.F., Masutani, M., Suzuki, H., and Caldecott, K.W. (2003). A requirement for PARP-1 for the assembly or stability of XRCC1 nuclear foci at sites of oxidative DNA damage. Nucleic Acids Res. *31*, 5526–5533.

Finn, R.D., Coggill, P., Eberhardt, R.Y., Eddy, S.R., Mistry, J., Mitchell, A.L., Potter, S.C., Punta, M., Qureshi, M., Sangrador-Vegas, A., et al. (2016). The Pfam protein families database: towards a more sustainable future. Nucleic Acids Res. *44* (D1), D279–D285.

Gibbs-Seymour, I., Oka, Y., Rajendra, E., Weinert, B.T., Passmore, L.A., Patel, K.J., Olsen, J.V., Choudhary, C., Bekker-Jensen, S., and Mailand, N. (2015). Ubiquitin-SUMO circuitry controls activated fanconi anemia ID complex dosage in response to DNA damage. Mol. Cell *57*, 150–164.

Gottschalk, A.J., Trivedi, R.D., Conaway, J.W., and Conaway, R.C. (2012). Activation of the SNF2 family ATPase ALC1 by poly(ADP-ribose) in a stable ALC1·PARP1·nucleosome intermediate. J. Biol. Chem. *287*, 43527–43532.

Hanahan, D., and Weinberg, R.A. (2011). Hallmarks of cancer: the next generation. Cell *144*, 646–674.

Hein, M.Y., Hubner, N.C., Poser, I., Cox, J., Nagaraj, N., Toyoda, Y., Gak, I.A., Weisswange, I., Mansfeld, J., Buchholz, F., et al. (2015). A human interactome in three quantitative dimensions organized by stoichiometries and abundances. Cell *163*, 712–723.

Helleday, T. (2011). The underlying mechanism for the PARP and BRCA synthetic lethality: clearing up the misunderstandings. Mol. Oncol. *5*, 387–393.

Isogai, S., Kanno, S., Ariyoshi, M., Tochio, H., Ito, Y., Yasui, A., and Shirakawa, M. (2010). Solution structure of a zinc-finger domain that binds to poly-ADP-ribose. Genes Cells *15*, 101–110.

Jackson, S.P., and Bartek, J. (2009). The DNA-damage response in human biology and disease. Nature *461*, 1071–1078.

Langelier, M.F., Planck, J.L., Roy, S., and Pascal, J.M. (2012). Structural basis for DNA damage-dependent poly(ADP-ribosyl)ation by human PARP-1. Science *336*, 728–732.

Mehrotra, P.V., Ahel, D., Ryan, D.P., Weston, R., Wiechens, N., Kraehenbuehl, R., Owen-Hughes, T., and Ahel, I. (2011). DNA repair factor APLF is a histone chaperone. Mol. Cell *41*, 46–55.

Muthurajan, U.M., Hepler, M.R., Hieb, A.R., Clark, N.J., Kramer, M., Yao, T., and Luger, K. (2014). Automodification switches PARP-1 function from chromatin architectural protein to histone chaperone. Proc. Natl. Acad. Sci. USA *111*, 12752–12757.

Pascal, J.M., and Ellenberger, T. (2015). The rise and fall of poly(ADP-ribose): an enzymatic perspective. DNA Repair (Amst.) *32*, 10–16.

Perina, D., Mikoč, A., Ahel, J., Ćetković, H., Žaja, R., and Ahel, I. (2014). Distribution of protein poly(ADP-ribosyl)ation systems across all domains of life. DNA Repair (Amst.) *23*, 4–16.

Ran, F.A., Hsu, P.D., Lin, C.Y., Gootenberg, J.S., Konermann, S., Trevino, A.E., Scott, D.A., Inoue, A., Matoba, S., Zhang, Y., and Zhang, F. (2013). Double nicking by RNA-guided CRISPR Cas9 for enhanced genome editing specificity. Cell *154*, 1380–1389.

Sharifi, R., Morra, R., Appel, C.D., Tallis, M., Chioza, B., Jankevicius, G., Simpson, M.A., Matic, I., Ozkan, E., Golia, B., et al. (2013). Deficiency of terminal ADP-ribose protein glycohydrolase TARG1/C6orf130 in neurodegenerative disease. EMBO J. *32*, 1225–1237.

Slade, D., Dunstan, M.S., Barkauskaite, E., Weston, R., Lafite, P., Dixon, N., Ahel, M., Leys, D., and Ahel, I. (2011). The structure and catalytic mechanism of a poly(ADP-ribose) glycohydrolase. Nature *477*, 616–620.

Timinszky, G., Till, S., Hassa, P.O., Hothorn, M., Kustatscher, G., Nijmeijer, B., Colombelli, J., Altmeyer, M., Stelzer, E.H., Scheffzek, K., et al. (2009). A macrodomain-containing histone rearranges chromatin upon sensing PARP1 activation. Nat. Struct. Mol. Biol. *16*, 923–929.

Xie, S., Mortusewicz, O., Ma, H.T., Herr, P., Poon, R.R., Helleday, T., and Qian, C. (2015). Timeless interacts with PARP-1 to promote homologous recombination repair. Mol. Cell *60*, 163–176.

Young, L.M., Marzio, A., Perez-Duran, P., Reid, D.A., Meredith, D.N., Roberti, D., Star, A., Rothenberg, E., Ueberheide, B., and Pagano, M. (2015). TIMELESS forms a complex with PARP1 distinct from its complex with TIPIN and plays a role in the DNA damage response. Cell Rep. *13*, 451–459.

Molecular Cell, Volume *62*

Supplemental Information

HPF1/C4orf27 Is a PARP-1-Interacting Protein
that Regulates PARP-1 ADP-Ribosylation Activity

Ian Gibbs-Seymour, Pietro Fontana, Johannes Gregor Matthias Rack, and Ivan Ahel

A

Organism	GenBank Accession no.	Function	Closest human homologue
Vertebrates	NP_775816	DNA repair	(APLF)
	NP_055696	DNA repair	(SNM1A)
	NP_001154816	DNA repair	(CHFR)
Drosophila melanogaster	NP_523465	DNA repair	TDP1
	NP_650455	DNA repair	APLF
	NP_649589	Unknown	C4orf27
Crassostrea gigas	XP_011423724	DNA repair	TDP1
	XP_011439503	DNA repair	APLF
	XP_011414049	Unknown	C4orf27
	XP_011433142	DNA repair	DNA ligase
	XP_011429878	(ADP-ribosyl)ation	PARP12
Dictyostelium discoideum	XP_628916	(ADP-ribosyl)ation	MACROD1
	XP_638785	(ADP-ribosyl)ation	TNKS2
	XP_641374	DNA repair	CHK2
	EAL61231	DNA repair	RAD17
	XP_637925	DNA repair	KU70
	XP_629877	DNA repair	UNG
	XP_641760	DNA repair	CHFR
Perkinsus marinum	XP_002765077	RNA recognition motifs (RRM) containing	CUGBP Elav-like family member 3
Emiliania huxleyi	XP_005762741	DNA repair	PNKP
	XP_005790986	Protease	Calpain-15
	XP_005780996	DNA repair	DNA ligase
Thecamonas trahens	XP_013754104	DNA repair	DNA ligase
	XP_013752674	Serine/threonine kinase	MAP3K7
	XP_013761104	DNA repair	Polβ
Capsaspora owczarzaki	XP_004365034	E2 ubiquitin-conjugating enzyme	UBE2Q1
	XP_004346784	Serine/threonine kinase	MAP3K7
	XP_004364110	DNA repair	DNA ligase
	XP_004364017	DNA repair	SNM1A

B

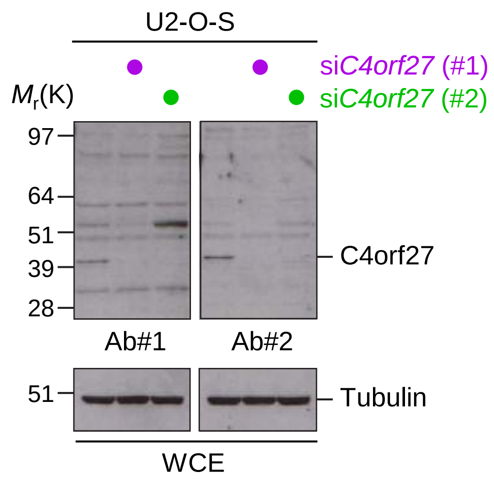

C

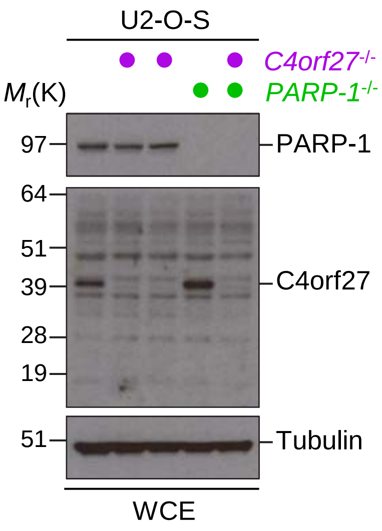

D

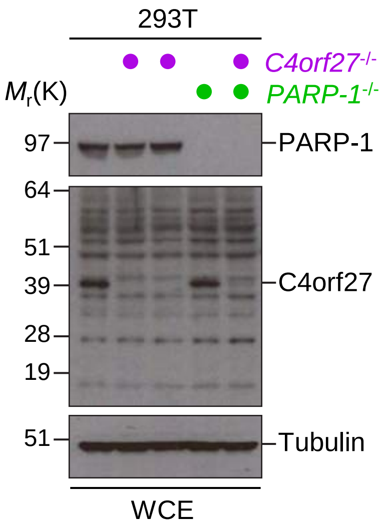

Figure S1 (related to Figure 1).

Identification of novel PBZ domain-containing proteins in eukaryotes

- **A.** Table showing PBZ-domain containing proteins in eukaryotes and their closest human orthologs.
- **B.** U2-O-S cells were transfected with siRNAs against C4orf27 for 72 h and whole cell extracts were analysed by immunoblotting with the indicated antibodies.
- **C.** U2-O-S cells with the indicated genetic backgrounds were generated using CRISPR/Cas9 and whole cell extracts were analysed by immunoblotting with the indicated antibodies.
- **D.** As for (C), but for 293T cells.

Gibbs-Seymour et al., Figure S2

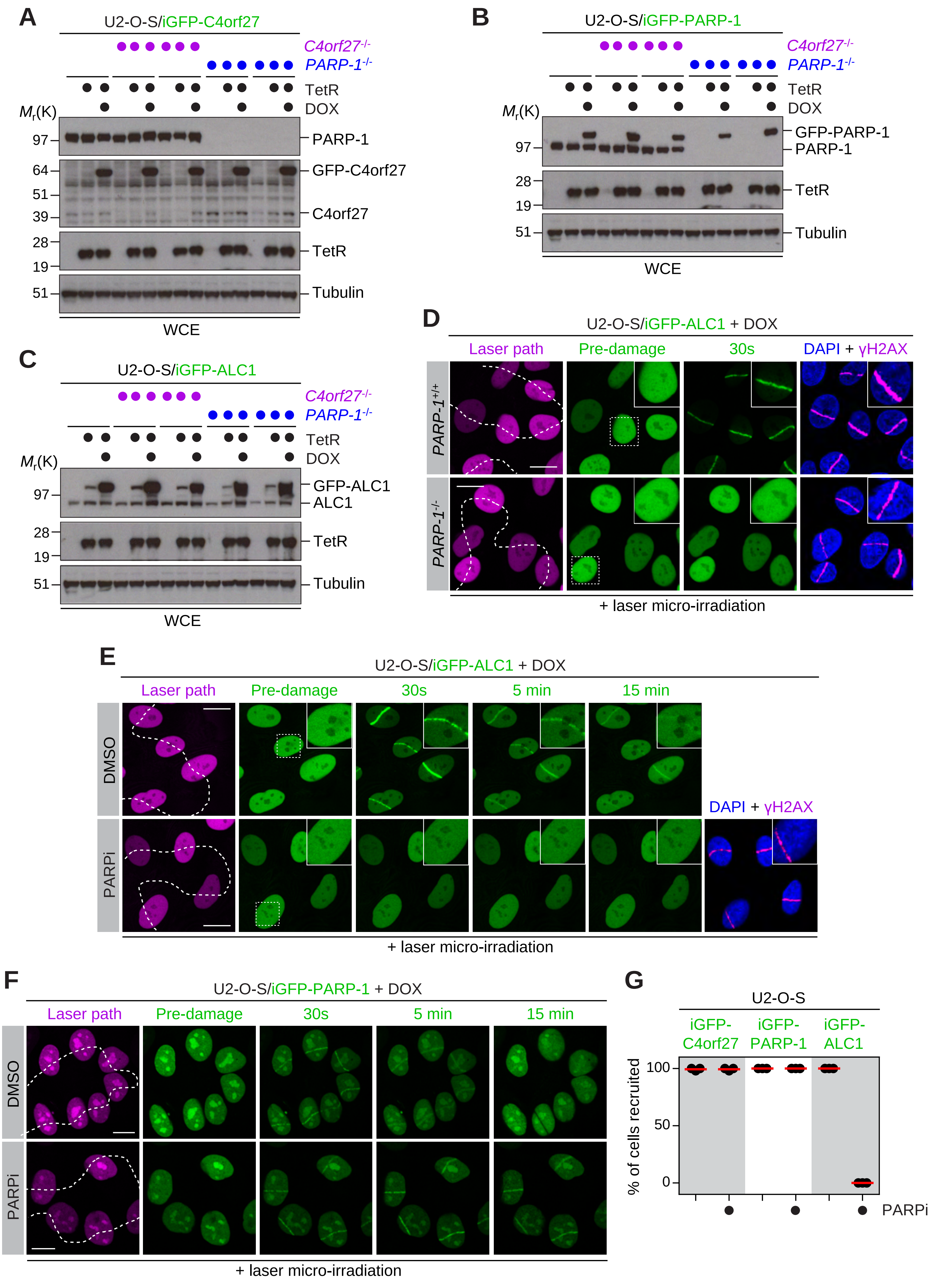

Figure S2 (related to Figure 2).

Generation of tools and laser micro-irradiation controls

- **A.** Indicated iGFP-C4orf27 cell lines (two independent clones for the *C4orf27*$^{-/-}$ and *PARP-1*$^{-/-}$ backgrounds) were treated with DOX for 24 h and whole cell extracts were analysed by immunoblotting with the antibodies shown.
- **B.** Indicated iGFP-PARP-1 cell lines (two independent clones for the *C4orf27*$^{-/-}$ and *PARP-1*$^{-/-}$ backgrounds) were treated with DOX for 24 h and whole cell extracts were analysed by immunoblotting with the antibodies shown.
- **C.** Indicated iGFP-ALC1 cell lines (two independent clones for the *C4orf27*$^{-/-}$ and *PARP-1*$^{-/-}$ backgrounds) were treated with DOX for 24 h and whole cell extracts were analysed by immunoblotting with the antibodies shown.
- **D.** U2-O-S/*PARP-1*$^{+/+}$ iGFP-ALC1 or U2-O-S/*PARP-1*$^{-/-}$ iGFP-ALC1 cells were induced with doxycycline (+DOX) for 24 h, subjected to laser micro-irradiation and imaged by live-cell microscopy at the indicated time. Cells were subsequently fixed and immunostained with γH2AX antibody. Scale bar, 10 µm.
- **E.** U2-O-S/iGFP-ALC1 cells were induced with DOX for 24 h, pre-treated with DMSO or PARPi for 1 h and then subjected to laser micro-irradiation and imaged by live-cell microscopy at the indicated time. Cells were subsequently fixed and immunostained with γH2AX antibody. Scale bar, 10 µm.
- **F.** U2-O-S/iGFP-PARP-1 cells were induced with DOX for 24 h, pre-treated with DMSO or PARPi for 1 h and then subjected to laser micro-irradiation and imaged by live-cell microscopy at the indicated time. Scale bar, 10 µm.
- **G.** Quantification of laser micro-irradiation experiments from Fig 2E and Fig S2E,F. Data represents mean±SEM from three biologically independent experiments. At least 50 cells were quantified in each experiment.

Gibbs-Seymour et al., Figure S3

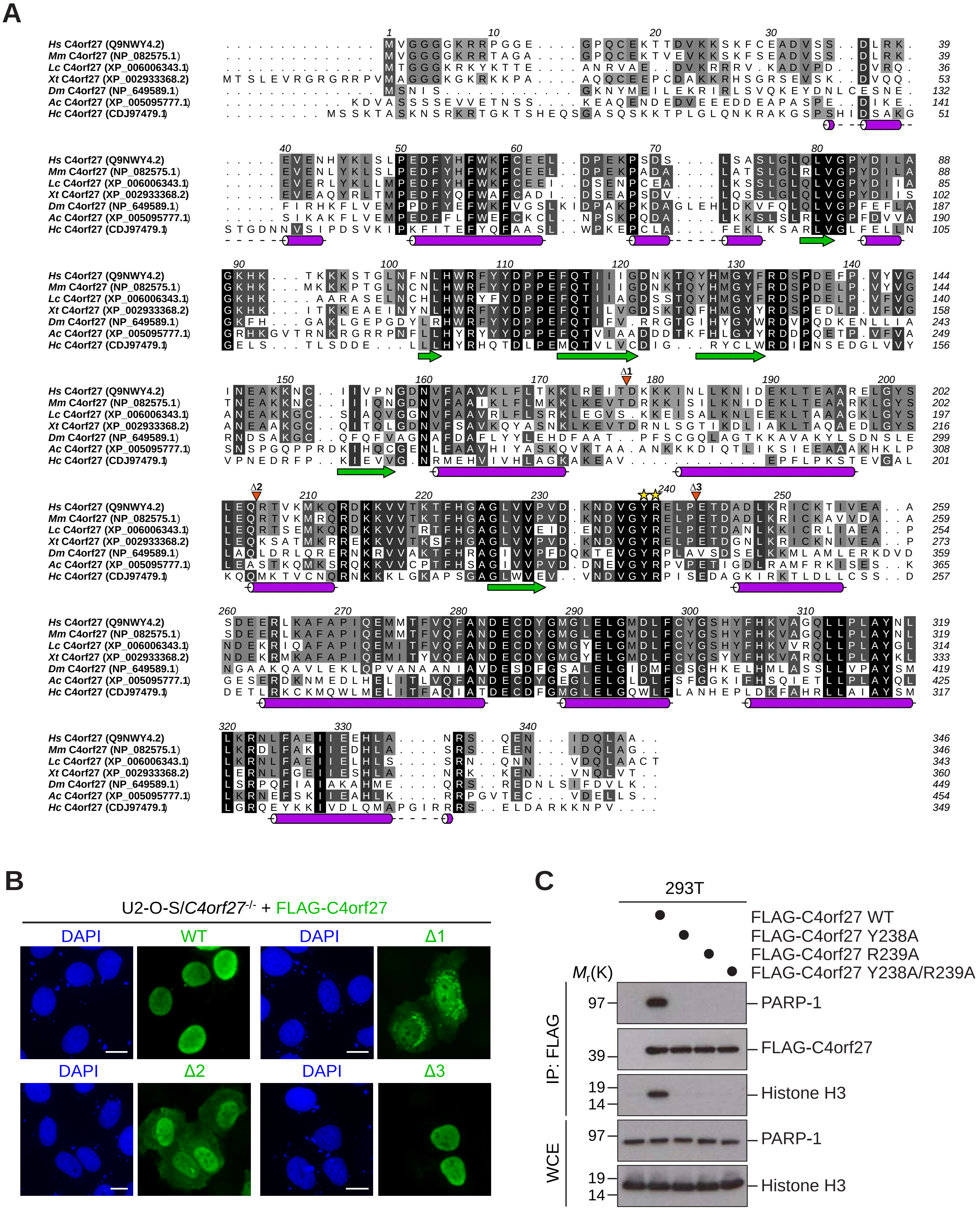

Figure S3 (related to Figure 3).

Identification of residues important for the C4orf27-PARP-1 interaction

A. Sequence alignment and secondary structure prediction of human C4orf27 and selected orthologs. Alignment representation was created using the ALINE software (Bond and Schuttelkopf, 2009). The locations of the Δ1, Δ2 and Δ3 deletion mutants are denoted by orange triangles and residues important for the PARP-1 interaction (Tyr238 and Arg239) are indictaed by yellow asterisks. *Hs, Homo sapiens*; *Mm, Mus musculus*; *Lc, Latimeria chalumnae*; *Xt*, *Xenopus tropicalis*; *Dm*, *Drosophila melanogaster*; *Ac, Aplysia californica*; *Hc*, *Haemonchus contortus*.

B. U2-O-S/*C4orf27*$^{-/-}$ cells were transfected with FLAG-C4orf27 WT or Δ1, Δ2 or Δ3 deletion mutants, fixed and immunostained with anti-FLAG antibody. Scale bar, 10 µm.

C. 293T cells were transfected with either FLAG-empty vector, FLAG-C4orf27 WT or point mutants Y238A, R239A or Y238A/R239A, subjected to FLAG immunoprecipitation and analysed by immunoblotting with the indicated antibodies.

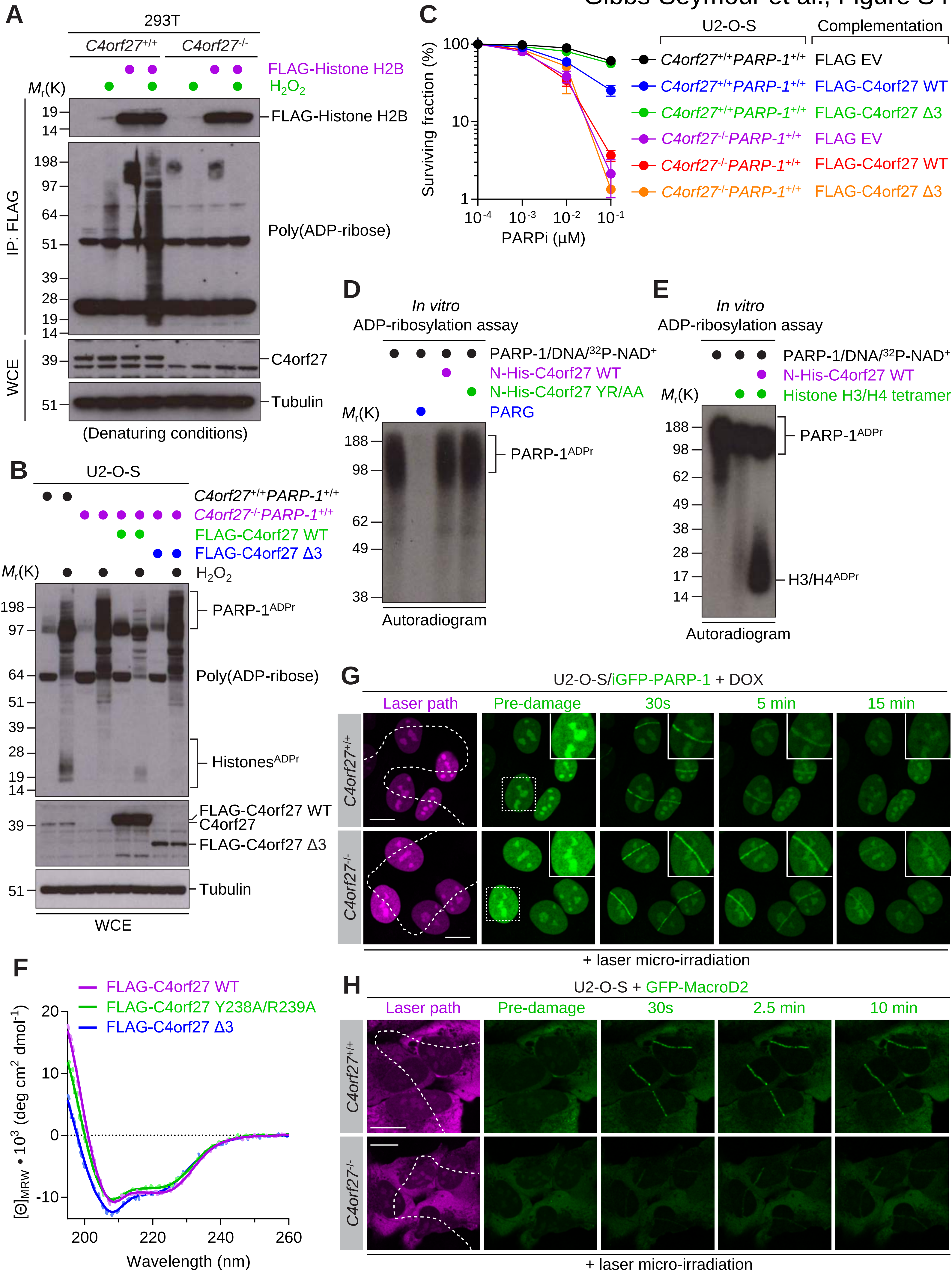
A
293T
C4orf27+/+
C4orf27-/-
FLAG-Histone H2B
H2O2
Mr(K)
IP: FLAG
FLAG-Histone H2B
Poly(ADP-ribose)
WCE
C4orf27
Tubulin
(Denaturing conditions)
B
U2-O-S
C4orf27+/+PARP-1+/+
C4orf27-/-PARP-1+/+
FLAG-C4orf27 WT
FLAG-C4orf27 Δ3
H2O2
PARP-1ADPr
Poly(ADP-ribose)
HistonesADPr
FLAG-C4orf27 WT
C4orf27
FLAG-C4orf27 Δ3
Tubulin
WCE
C
U2-O-S
Complementation
Surviving fraction (%)
PARPi (μM)
C4orf27+/+PARP-1+/+ FLAG EV
C4orf27+/+PARP-1+/+ FLAG-C4orf27 WT
C4orf27+/+PARP-1+/+ FLAG-C4orf27 Δ3
C4orf27-/-PARP-1+/+ FLAG EV
C4orf27-/-PARP-1+/+ FLAG-C4orf27 WT
C4orf27-/-PARP-1+/+ FLAG-C4orf27 Δ3
D
In vitro
ADP-ribosylation assay
PARP-1/DNA/32P-NAD+
N-His-C4orf27 WT
N-His-C4orf27 YR/AA
PARG
PARP-1ADPr
Autoradiogram
E
In vitro
ADP-ribosylation assay
PARP-1/DNA/32P-NAD+
N-His-C4orf27 WT
Histone H3/H4 tetramer
PARP-1ADPr
H3/H4ADPr
Autoradiogram
F
FLAG-C4orf27 WT
FLAG-C4orf27 Y238A/R239A
FLAG-C4orf27 Δ3
[Θ]MRW • 10^3 (deg cm^2 dmol^-1)
Wavelength (nm)
G
U2-O-S/iGFP-PARP-1 + DOX
Laser path
Pre-damage
30s
5 min
15 min
C4orf27+/+
C4orf27-/-
+ laser micro-irradiation
H
U2-O-S + GFP-MacroD2
Laser path
Pre-damage
30s
2.5 min
10 min
C4orf27+/+
C4orf27-/-
+ laser micro-irradiation

Figure S4 (related to Figure 4).

C4orf27 promotes *in trans* ADP-ribosylation of histones and prevents PARP-1 hyper auto-modification

- **A.** 293T/*C4orf27*$^{+/+}$ or 293T/*C4orf27*$^{-/-}$ cells were transfected with FLAG-empty vector, or FLAG-histone H2B, treated with either vehicle or H_2O_2 (1 μM) for 10 min, lysed under denaturing conditions and then subjected to FLAG immunoprecipitation and analysed by immunoblotting with the indicated antibodies.
- **B.** U2-O-S cells with the indicated genetic backgrounds were treated with H_2O_2 (1 μM) for 10 min and whole cell extracts were analysed by immunoblotting with the indicated antibodies. ADP-ribosylated PARP-1 (PARP-1^{ADPr}) and histones (Histones^{ADPr}) are indicated.
- **C.** U2-O-S/*C4orf27*$^{+/+}$ or U2-O-S/*C4orf27*$^{-/-}$ cells complemented with empty vector, FLAG-C4orf27 WT or FLAG-C4orf27 Δ3 were treated with various doses of PARPi and analysed for clonogenic survival. Data represents mean±SEM from three biologically independent experiments using technical triplicates per datapoint.
- **D.** Recombinant PARP-1 was incubated with DNA and ^{32}P-NAD^{+} to produce ADP-ribosylated PARP-1, before the addition of PARP inhibitor to stop the reaction. Recombinant PARG or C4orf27 was then added to the reaction and products were analysed by SDS-PAGE and autoradiography.
- **E.** Recombinant PARP-1 and N-His-C4orf27 were incubated with recombinant histone H3/H4 tetramer and reactions were initiated by addition of DNA and ^{32}P-NAD^{+}. Reaction products were subsequently analysed by SDS-PAGE and autoradiography.
- **F.** CD analysis of recombinant C4orf27 wild type, Y238A/R239A and Δ3. CD spectra (195-260nm) were obtained at 20°C and buffer corrected. Dashed lines represent

accumulated spectra measurements (*n*=15) and solid lines smoothed spectra using the Savitzky-Golay algorithm.

G. U2-O-S/*C4orf27*$^{+/+}$ iGFP-PARP-1 or U2-O-S/*C4orf27*$^{-/-}$ iGFP-PARP-1 cells were induced with doxycycline (+DOX) for 24 h, subjected to laser micro-irradiation and imaged by live-cell microscopy at the indicated time. Scale bar, 10 μm.

H. U2-O-S/*C4orf27*$^{+/+}$ or U2-O-S/*C4orf27*$^{-/-}$ cells were transfected with GFP-MacroD2, subjected to laser micro-irradiation and imaged by live-cell microscopy at the indicated time. Scale bar, 10 μm.

Supplemental Experimental Procedures

Lentiviral vectors and RNAi

To generate lentiviral vectors expressing GFP-tagged alleles in a doxycycline-dependent manner, full-length human C4orf27, PARP-1 and ALC1 cDNAs were each cloned into the pENTR4-GFP-C1 vector (Addgene plasmid #17396) (Campeau et al., 2009). These entry vectors were then used in an LR reaction together with the Gateway compatible pLenti-CMV/TO-Hygro destination vector (Addgene plasmid #17291), to generate pLenti-CMV/TO-Hygro-GFP-C4orf27, pLenti-CMV/TO-Hygro-GFP-PARP-1 and pLenti-CMV/TO-Hygro-GFP-ALC1. pLenti-CMV-TetR-Blast was used to generate TetR containing lentivirus (Addgene plasmid #17492). To generate lentiviral vectors expressing FLAG-tagged alleles in a constitutive manner, full length C4orf27 or the Δ3 deletion were cloned into pENTR4-FLAG (Addgene plasmid # 17423). These entry vectors were then used in an LR reaction together with the Gateway compatible pLenti-CMV-Hygro destination vector (Addgene plasmid #17454), to generate pLenti-CMV-Hygro-FLAG-C4orf27 WT or Δ3. For protein expression, C4orf27, PARP-1 and PARP-2 were cloned into the pET-28a(+) vector (Novagen) or pGEX-4T1 vector (GE Healthcare). All constructs described above were verified by Sanger sequencing. siRNAs against the C4orf27 open reading frame were purchased from Ambion (s29882 and s29883). Sequence information for each C4orf27 siRNA is available upon request. The control siRNA sequence was published previously (Gibbs-Seymour et al., 2015).

Lentivirus production

All lentiviruses were generated using the pCMV-dR8.2 dvpr and pCMV-VSV-G vectors, both kind gifts from Dr Bob Weinberg (Addgene plasmids #8455 and #8454, respectively) (Stewart et al., 2003), together with either pLenti-CMV-TetR-Blast, pLenti-CMV/TO-Hygro-GFP-C4orf27, pLenti-CMV/TO-Hygro-GFP-PARP-1 or pLenti-CMV/TO-Hygro-GFP-ALC1 vectors, as described above.

Immunochemical methods

Primary antibodies used in this study included: mouse monoclonals to ALC1 (Abcam), FLAG-M2-HRP (Sigma), γH2AX (Millipore), 6xHistidine (Clontech), Myc (Clontech), TetR (MoBiTec); rabbit polyclonals to Histone H2A (Abcam), Histone macroH2A1.1 (Abcam), Histone H3 (Millipore), Ku70 (Abcam), PARP-1 (Abcam), poly(ADP-ribose) (Trevigen), poly(ADP-ribose) (Enzo), Tubulin (Abcam), XRCC1 (Abcam); goat polyclonal to GST-HRP (Abcam). Rabbit polyclonal antibodies against C4orf27 were generated using full-length His-tagged C4orf27. Co-immunoprecipitation of protein complexes from cells followed by immunoblotting analysis was performed essentially as described previously (Gibbs-Seymour et al., 2015; Mehrotra et al., 2011). Briefly, for co-immunoprecipitation of native complexes, cells were lysed in buffer containing 50 mM Tris-HCl pH 8, 1% Triton X-100, 100 mM NaCl, Benzonase nuclease (Sigma), 1 μM PARP inhibitor (olaparib), 1 μM PARG inhibitor (ADP-HPD), 1 mM DTT, protease and phosphatase inhibitors. Lysates were clarified and added to FLAG-M2 affinity gel for 15 min whilst rotating at 4°C. Beads were washed several times with lysis buffer and eluted with LDS sample buffer (Thermo Fisher).

Mass spectrometry-based analysis of C4orf27 interactors

Protein complexes were immunopurified from 239T cells as described above. After purification, FLAG-C4orf27 or FLAG-empty vector immunoprecipitates were washed several times with lysis buffer without any Triton X-100. Beads from three independent experiments were then subjected to an on-bead tryptic digest using published FASP protocols (Wisniewski et al., 2009). Mass spectrometry was carried out essentially as described previously, using a Q Exactive mass spectrometer (Thermo, Hemel Hempstead) coupled to a Dionex Ultimate 3000 RSLCnano system (Palazzo et al., 2015). Mass spectra were searched against the Human complete proteome database using the Andromeda algorithm through MaxQuant (version 1.5.2.8) (Cox and Mann, 2008). Missing values were substituted with the minimum for each

sample and label-free quantification normalised protein intensities were log2 transformed (Cox et al., 2014). An empirical Bayes moderated t-statistics for paired samples, as implemented in the R limma package, was applied to identify proteins with a significant difference in abundance. The Benjamini and Hochberg multiple testing correction was applied to control false discovery rate.

Immunofluorescence, laser micro-irradiation and microscopy

Immunofluorescence protocols were as described previously (Gibbs-Seymour et al., 2015). Cells were pre-sensitised for 24 h before laser micro-irradiation using 10 μM BrdU (Sigma) and the media was changed to Live Cell Imaging Solution (Thermo Fisher) before laser micro-irradiation and imaging. Laser micro-irradiation was performed on an Olympus Fluoview FV1200 confocal microscope equipped an inverted IX83 motorised stage with a 37ºC humidified chamber and 60x/1.40 oil UPlanSApo objective and 405nm laser. Confocal microscopy was performed on the same microscope. Standard wide-field microscopy was performed on the Olympus BX61 microscope system, equipped with a 40x/0.75 dry objective, a CoolSNAP HQ2 camera (Roper Scientific) and MetaMorph 7.5 imaging software (GE Healthcare).

Colony formation assays

For colony formation assays, cells were plated at low densities in the presence of the indicated doses of methyl methanesulfonate (MMS). Cells were subsequently fixed and stained with crystal violet after 10 days for 293T cells. The surviving fraction at each dose was calculated after normalisation to the plating efficiency of untreated samples. For PARP inhibitor sensitivity assays, U2-O-S cells were plated in varying molarities of olaparib (stock dissolved in DMSO). Media containing fresh olaparib or vehicle was replaced every 72 h and cells were fixed and processed after 12 days as described above.

GST pull-down assays

Pull-down assays were performed essentially as described previously (Rack et al., 2015), except the TZNK/D/T buffer contained 400 mM KCl.

Circular Dichroism

Circular dichroism (CD) was carried out at 20°C using a Jasco J-815 spectropolarimeter (JASCO analytical instruments) and a 1 mm path length CD cell (Starna Scientific). Sample were purified as described above, followed by size exclusion chromatography using a HiLoad Superdex 75pg column (GE Healthcare) and 25 mM sodium phosphate pH 8 and 150 mM NaCl. This was followed by a two-step dialysis against CD buffer, 25 mM sodium phosphate pH 8 and 150 mM NaF. Samples were diluted with CD buffer to 2.5 µM before measurement. All data were background corrected and have been converted from raw ellipticity to molar residue ellipticity ($[\Theta]_{MRW}$) according to Equation 1,

$$[\Theta]_{\mathrm{MRW}} = \frac{\Theta}{10 \cdot \mathrm{c_r} \cdot \ell} \qquad (1)$$

where Θ is the CD signal of the sample in millidegrees; ℓ is the path length of the cell in centimetres; and c_r is the mean residue concentration in molar.

Supplemental References

Bond, C.S., and Schuttelkopf, A.W. (2009). ALINE: a WYSIWYG protein-sequence alignment editor for publication-quality alignments. Acta Crystallogr D Biol Crystallogr *65*, 510-512.
Campeau, E., Ruhl, V.E., Rodier, F., Smith, C.L., Rahmberg, B.L., Fuss, J.O., Campisi, J., Yaswen, P., Cooper, P.K., and Kaufman, P.D. (2009). A versatile viral system for expression and depletion of proteins in mammalian cells. PloS one *4*, e6529.
Cox, J., Hein, M.Y., Luber, C.A., Paron, I., Nagaraj, N., and Mann, M. (2014). Accurate proteome-wide label-free quantification by delayed normalization and maximal peptide ratio extraction, termed MaxLFQ. Mol Cell Proteomics *13*, 2513-2526.
Cox, J., and Mann, M. (2008). MaxQuant enables high peptide identification rates, individualized p.p.b.-range mass accuracies and proteome-wide protein quantification. Nat Biotechnol *26*, 1367-1372.
Gibbs-Seymour, I., Oka, Y., Rajendra, E., Weinert, B.T., Passmore, L.A., Patel, K.J., Olsen, J.V., Choudhary, C., Bekker-Jensen, S., and Mailand, N. (2015). Ubiquitin-SUMO circuitry controls activated fanconi anemia ID complex dosage in response to DNA damage. Mol Cell *57*, 150-164.
Mehrotra, P.V., Ahel, D., Ryan, D.P., Weston, R., Wiechens, N., Kraehenbuehl, R., Owen-Hughes, T., and Ahel, I. (2011). DNA repair factor APLF is a histone chaperone. Mol Cell *41*, 46-55.
Palazzo, L., Thomas, B., Jemth, A.S., Colby, T., Leidecker, O., Feijs, K.L., Zaja, R., Loseva, O., Puigvert, J.C., Matic, I., *et al.* (2015). Processing of protein ADP-ribosylation by Nudix hydrolases. Biochem J *468*, 293-301.
Rack, J.G., Morra, R., Barkauskaite, E., Kraehenbuehl, R., Ariza, A., Qu, Y., Ortmayer, M., Leidecker, O., Cameron, D.R., Matic, I., *et al.* (2015). Identification of a Class of Protein ADP-Ribosylating Sirtuins in Microbial Pathogens. Mol Cell *59*, 309-320.
Stewart, S.A., Dykxhoorn, D.M., Palliser, D., Mizuno, H., Yu, E.Y., An, D.S., Sabatini, D.M., Chen, I.S., Hahn, W.C., Sharp, P.A., *et al.* (2003). Lentivirus-delivered stable gene silencing by RNAi in primary cells. RNA *9*, 493-501.
Wisniewski, J.R., Zougman, A., Nagaraj, N., and Mann, M. (2009). Universal sample preparation method for proteome analysis. Nature methods *6*, 359-362.
